# Conserved and Divergent Features of Adult Neurogenesis in Zebrafish

**DOI:** 10.3389/fcell.2020.00525

**Published:** 2020-06-30

**Authors:** Miriam Labusch, Laure Mancini, David Morizet, Laure Bally-Cuif

**Affiliations:** ^1^Zebrafish Neurogenetics Unit, Institut Pasteur, UMR 3738, CNRS, Team Supported by the Ligue Nationale Contre le Cancer, Paris, France; ^2^Sorbonne Université, Collège Doctoral, Paris, France

**Keywords:** neural stem cell, zebrafish, pallium, adult neurogenesis, quiescence, radial glia, cell fate choice decisions

## Abstract

Adult neurogenesis, i.e., the generation of neurons from neural stem cells (NSCs) in the adult brain, contributes to brain plasticity in all vertebrates. It varies, however, greatly in extent, location and physiological characteristics between species. During the last decade, the teleost zebrafish (*D. rerio*) was increasingly used to study the molecular and cellular properties of adult NSCs, in particular as a prominent NSC population was discovered at the ventricular surface of the dorsal telencephalon (pallium), in territories homologous to the adult neurogenic niches of rodents. This model, for its specific features (large NSC population, amenability to intravital imaging, high regenerative capacity) allowed rapid progress in the characterization of basic adult NSC features. We review here these findings, with specific comparisons with the situation in rodents. We specifically discuss the cellular nature of NSCs (astroglial or neuroepithelial cells), their heterogeneities and their neurogenic lineages, and the mechanisms controlling NSC quiescence and fate choices, which all impact the neurogenic output. We further discuss the regulation of NSC activity in response to physiological triggers and non-physiological conditions such as regenerative contexts.

Adult neurogenesis, first identified as such in birds (Goldman and Nottebohm, 1983), has been documented in all vertebrate species studied (Altman and Das, 1965; Eriksson et al., 1998; Byrd and Brunjes, 2001; Suh et al., 2007). The persistence of neuronal production in the adult brain is the product of specialized neural precursor cells, the neural stem cells (NSCs). In rodents, newly-born neurons are physiologically important for the plasticity of specific circuits, notably involved in learning and memory, and impaired adult neurogenesis can correlate with emotional disorders (Anacker and Hen, 2017; Jorgensen, 2018; Toda et al., 2019). NSCs have also been postulated to be at the origin of some brain tumors (Fan et al., 2019; Matarredona and Pastor, 2019). The fundamental importance of NSCs stimulated an explosive research field during the last 20-years, and, more recently, the development of a new study model: the zebrafish adult brain. The large amount of adult NSCs in this system, their widespread distribution and varied properties, and their reactivity toward regeneration, all propelled the zebrafish model to the forefront of adult NSC research, as a complementary and synergistic model to rodents (Anand and Mondal, 2017; Lindsey et al., 2018; Zambusi and Ninkovic, 2020). The time to reach sexual maturity in zebrafish (3 months) and the adult lifespan also approximate those of mouse, allowing to draw direct temporal parallels. With specific focus on NSCs of the dorsal telencephalon, we will review here these different attributes, stressing the contribution of the zebrafish model to understand basic NSC properties such as their lineages, quiescence, fate choices, heterogeneities, population behavior and their physiological and pathological recruitment.

## Neural Stem Cells: a Variety of Progenitor Cell Subtypes Drive Neurogenesis in the Adult Zebrafish Central Nervous System

### Active Neurogenesis From Multiple Neurogenic Niches

**FIGURE 1:**
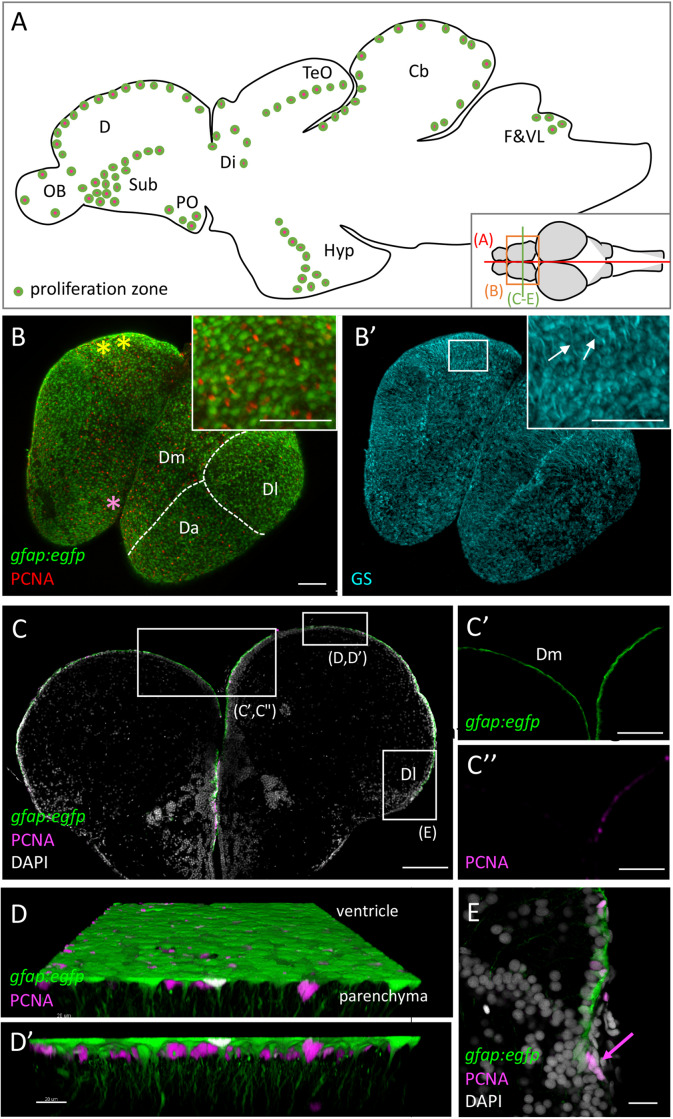
Progenitor cells in the zebrafish adult brain at 3 months-post-fertilization (mpf). **(A)** Scheme of a mid-sagittal section (anterior left) showing the localization of proliferation zones (colored dots) (Adolf et al., 2006; Grandel et al., 2006). **(B,B’)** Dorsal view of a whole-mount telencephalon from a *gfap:egfp* transgenic animal, processed in triple immunohistochemistry for GFP, PCNA **(B)**, and GS **(B’)**. Anterior is bottom left. Note the continuous layer of progenitor cells visible from the dorsal surface. Pallial territories are indicated by the dotted lines (see Dray et al., 2015). Yellow stars indicate the location of the territory homologous to the hippocampus (Ganz et al., 2015, and see Rodríguez et al., 2002 in goldfish), and the pink star the territory homologous to the amygdala (von Trotha et al., 2014). Anti-GS immunohistochemistry **(B’)** permits to see basal RG processes (arrows). **(C–E)** Cross-section of a telencephalon from a *gfap:egfp* transgenic animal, processed in double immunohistochemistry for GFP and PCNA and counter-stained with DAPI **(C)** and high magnifications of the domains boxed **(C’,C”,E).** In addition, a high magnification view of the ventricular zone of Dm is shown (**D,D’**) in 3D (Imaris software) to appreciate radial glial cell morphology. **(E)** Focus on NE progenitors at the pallial edge (arrow). Scale bars: **(B,B’,C)** 100 mm; **(C’,C”)** 30 mm; **(D,D’)** 20 mm; **(E)** 50 mm. Cb, cerebellum; D, dorsal part of the telencephalon (pallium) (Da: anterior part of D, Dm: medial part of D; Dl, lateral part of D); Di, diencephalon; F&VL, facial and vagal lobes; Hyp, hypothalamus; OB, olfactory bulb; PO, preoptic area; TeO, tectum opticum.

The persistent and widespread neurogenic activity of the zebrafish adult brain was first recognized using classical tracing methods employing thymidine analogs: 16 proliferation domains, present across all brain subdivisions, proved to be at the origin of neurons within a few weeks of chase (Figure 1A; Adolf et al., 2006; Grandel et al., 2006). Using similar approaches, physiologically silent but activatable neural progenitors were also identified in the adult zebrafish spinal cord (Reimer et al., 2009). These constitutive and facultative neurogenic niches raised important interest. Indeed, by their variety, they permit comprehensive comparisons of neurogenic progenitor identities and properties, and of neurogenesis modes, in the adult vertebrate central nervous system.

We will focus in this review on adult neurogenesis in the zebrafish telencephalon, which hosts the territories homologous to two main neurogenic niches of adult rodents: the sub-ependymal zone of the lateral ventricle (SEZ) and the sub-granular zone (SGZ) of the dentate gyrus of the hippocampus [for completeness on other territories, the reader is referred to other recent reviews or articles (Than-Trong and Bally-Cuif, 2015; Anand and Mondal, 2017; Lindsey et al., 2018)]. Following a process of eversion, likely involving both morphogenetic cell shape changes and anisotropic growth, the ventricle of the zebrafish dorsal telencephalon (pallium) becomes exposed dorsally, covered by an enlarged choroid plexus, with its dorsal midline flipped to lateral positions (Folgueira et al., 2012). This results in a medio-lateral inversion of homologous pallial territories between zebrafish and mammals. A tentative correspondence, based on ontogenetic and functional grounds, has been proposed (von Trotha et al., 2014; Dray et al., 2015; Ganz et al., 2015).

### Neural Stem Cells and Neural Progenitors in the Adult Zebrafish Telencephalon

A variety of genetic and non-genetic tracing methods (Table 1), coupled with precise immunohistochemical or molecular characterizations, identified several neural progenitor subtypes in the adult zebrafish telencephalon. Some of them, notably radial glia (RG) of the pallium, are considered NSCs (discussion of the “NSC” versus “neural progenitor” nomenclature in Box 1, and see below).

**TABLE 1:** Tracing neural progenitors and/or their progeny in the adult zebrafish telencephalon.

Method	Principle	Output (and limitations)	Princeps publications
Thymidine analogs	These compounds (BrdU, CldU, EdU) incorporate into the DNA of cycling cells during the S phase. They are revealed by immuno-histochemistry or click-chemistry.	Labeling of dividing cells only (thus low efficiency to label dormant cells). Identification of the progeny cells becoming post-mitotic or dividing infrequently post-labeling (dilution of the label at each division round, so rapidly dividing progeny cells are lost). Detection of cells dividing infrequently, when detected together with a proliferation marker (PCNA, MCM proteins) after a chase (“label retention assay”).	Adolf et al., 2006; Grandel et al., 2006; Pellegrini et al., 2007
Retroviruses	Cells with ventricular contact are infected upon intra-ventricular injection of the viral suspension. Following infection, the genetic material carried by the virus is reverse transcribed and integrates into the host cell genome.	Integration into dividing cells for simple retroviruses, and into non-dividing cells as well for lentiviruses, the genetic material of which can cross nuclear pores. Permanent labeling of the progenitor and its progeny. Cell specificity of expression can be achieved using specific promoters.	Rothenaigner et al., 2011
DNA electroporation or lipofection	Cells with ventricular contact are targeted upon intra-ventricular injection of the virus suspension and electroporation. DNA remains episomal.	Cell specificity of expression can be achieved using specific promoters. Labeling is transmitted to progeny cells but is *a priori* not permanent. Bias toward targeting cells with a large apical surface.	Chapouton et al., 2010; Alunni et al., 2013
Conditional Cre-lox-mediated genetic tracing	Double transgenic animals (driver-reporter) are used. Expression of Cre-ER is driven from the driver transgene by neural progenitor-specific promoters, and nuclear translocation is temporally controlled by tamoxifen treatment. It recombines the reporter transgene at LoxP sites to express a reporter, usually driven by a ubiquitous promoter.	Cell specificity of the recombination is achieved using specific promoters (so far: *her4.1*; *gfap*; *nestin*); these promoters may not recapitulate the endogenous pattern in all lines, and need to drive strong expression for recombination to be efficient. Labeling is permanent in the progenitor and all its progeny cells. Various extents of recombination can be used (from clonal to full).	Kroehne et al., 2011
Tet-rtTA-mediated genetic tracing	Double transgenic animals (driver-reporter) are used. Expression of Tet is driven from the driver transgene by neural progenitor-specific promoters, and its activity is temporally controlled by doxycylin treatment. It then activates the reporter transgene.	Cell specificity of induction is achieved using specific promoters (so far: *her4.1*). Labeling is transient in the progenitor following arrest of the doxycycline treatment. If the reporter protein is fused with a histone (e.g., H2B), it will be diluted in the progenitor cell upon division, but stably maintained in post-mitotic cells generated soon after induction, hence also serving as a birth dating method; like with thymidine analogs, rapidly dividing progeny cells will be lost by label dilution. Various extents of induction can be used (from clonal to full); full inductions can also be used to track non-dividing progenitor cells that retain the label (although with caution, as expression levels at induction may be variable).	Furlan et al., 2017
Intravital imaging	2P: Semi-transparent adult animals (*casper* or *nacre*) are used, anesthetized and imaged using 2P microscopy. Progenitor cells are tracked using specific transgenic reporter backgrounds or following reporter electroporation. 3P: transgenic *casper* adults are used, anesthetized and imaged using 3P microscopy.	Individual progenitor cells can be tracked over some weeks. Tracking of progeny cells is transient as they leave the progenitor niche to reach deep parenchymal areas. Only applicable so far to the dorsal-most pallial areas (Da, Dm). Individual progenitors can be imaged, as well as cells located much deeper in the parenchyma (at least 200 mm below the NSC layer), e.g., neurons. Howerver the method has not been used yet for repetitive imaging.	Barbosa et al., 2015a; Dray et al., 2015; Guesmi et al., 2018

**Box 1 |**

Neural Stem Cells and neural progenitors.

By definition, **stem cells** are individual cells endowed with long-term self-renewal and at least bi-potency. This initial definition is in line with a classical scheme where a stem cell upon division generates another stem cell and a differentiated progeny. However, clonal tracing in a number of adult stem cell systems rather supports a model where stem cells are self-renewing and bi-potent at the population level, choosing stochastically between balanced numbers of amplifying, asymmetric or differentiative divisions. This is no exception in the adult brain where several studies, both in mouse and zebrafish, are compatible, at least in part, with such “population asymmetry” ensuring both neural stem cell maintenance and neuronal production. These converging observations suggest to revise the strict definition of a neural stem cell toward that of neural stem cell population(s), characterized by their capacity, as a whole, to maintain themselves and generate neurons and/or post-mitotic glial cells. In the zebrafish adult pallium, these properties are met by radial glial cells (although to varying degrees). One can further distinguish constitutive and facultative neural stem cells (or population), the former being active physiologically and the latter being normally silent but becoming active, e.g., upon lesion (as is for example the case in the zebrafish spinal cord). The term “**neural progenitor**” is generally used more broadly, (i) to mention progenitors that are further committed along the neurogenesis lineage than neural stem cells (for example, the “activated neural progenitors” -NPs- of the zebrafish adult pallium or the equivalent “transit amplifying progenitors” -TAPs- of adult mouse neurogenic niches), (ii) to refer to neurogenic cells whose self-renewal potential has not been clearly assessed, (iii) or to jointly refer to all cells with neurogenic capacity (for example, NSCs + NPs).

#### Pallial Radial Glial Cells Are Molecularly and Cellularly Similar to Rodent Adult NSCs

Pallial RG are organized as a tight monolayer with their cell bodies lining the pallial ventricle. They exhibit overt apico-basal polarity, exposing a small apical membrane domain to the cerebrospinal fluid and extending a long and highly branched basolateral process across the pallial parenchyma (Figures 1C–D’). Pallial RG express astroglial markers (Glial Fibrilary Acidic Protein - gfap-, Brain Lipid-Binding Protein - blbp-, Nestin, Glutamine Synthetase -GS-) as well as S100β, which highlights NSCs and ependymal cells in rodents, and Aromatase B (Adolf et al., 2006; Grandel et al., 2006; Pellegrini et al., 2007; März et al., 2010a). Parenchymal astrocytes are absent from the adult zebrafish pallium before aging (Ogino et al., 2016); this observation and the expression of factors encoding astrocytic function in RG (GS, and the glutamate transporters Glast and Glt1) suggest that pallial RG serve the function of parenchymal astrocytes, and extend this function into the parenchyma via their basal process. Overall, the morphology and astroglial markers of pallial RG resemble those of adult NSCs in the mouse SEZ and SGZ (Than-Trong and Bally-Cuif, 2015). They also morphologically resemble radial glial cells of the developing mouse cortex, but differ from these in several other aspects such as their proliferation potential and activity, and their transcriptome (Götz et al., 2016) (and see below). In detail, distinct morphologies were described among pallial RG depending on their location (März et al., 2010a). To date, these differences have not been related to functional (whether astrocytic or stem) properties.

Like adult mouse NSCs, pallial RG co-express progenitor markers, such as the transcription factors Sox2, Hey1, and Her4 (mouse Hes5) (Kroehne et al., 2011; Than-Trong et al., 2018; Than-Trong et al., 2020). Of these, only the function of Hey1 was tested to date in zebrafish, and shown to be necessary for the maintenance of progenitor potential *in vivo* (Than-Trong et al., 2018). Hes5 (together with the related Hes factor Hes1) as well as Sox2 are, however, necessary for NSC maintenance in adult mouse (Ehm et al., 2010; Boareto et al., 2017; Sueda et al., 2019), and are likely to play a similar role in zebrafish.

At any given time, approximately 5% of pallial RG are found within the cell cycle (i.e., express the proliferation parkers PCNA or MCM2/5; these cells are referred to as “activated”). The remaining 95% are out of the cell cycle and interpreted as quiescent (see below) (Chapouton et al., 2010; März et al., 2010a). This interpretation as well as the self-renewal and neurogenic potential of the pallial RG population are supported by a number of converging arguments, including: (i) tracing assays demonstrating that individual RGs can oscillate between the activated and quiescent states, (ii) pharmacological assays or experimental injuries demonstrating that all, or most, RG can be brought into the activated state, (iii) genetic tracing identifying RG progeny at the individual and populational levels (Chapouton et al., 2010; März et al., 2010a; Kroehne et al., 2011; Alunni et al., 2013; Than-Trong et al., 2020). The latter experiments are particularly important as they demonstrate that *her4*-positive RG generate both pallial neurons and persisting pallial RG that are themselves neurogenic. Thus, at least at the population level, *her4*-positive RG act as NSCs. For comparison, RG of the developing mouse cortex do not exhibit quiescence phase, and their neurogenic activity is limited to the embryonic period (Götz et al., 2016).

#### Non-glial Pallial Progenitors

In addition, non-glial neural progenitors (NPs) (negative for astroglial markers and for *her4*) are present interspersed among RGs along the pallial ventricle (Ganz et al., 2010; März et al., 2010a). NPs are identified as progenitors according to their expression of Sox2, the fact that around 50% of them co-express proliferation markers at any time, and their neurogenic fate (assessed by retroviral tracing at short term) (Rothenaigner et al., 2011; Than-Trong et al., 2020). Cre-lox tracing with short chase times indicates these cells originate from pallial RG (Than-Trong et al., 2020). This population, however, lacks specific markers and to date was only relatively superficially analyzed. It is possibly heterogeneous, and in particular the properties of Sox2+;PCNA- cells have not been directly defined. NPs are classically considered equivalent to the Transit Amplifying Progenitors (TAPs) described in mouse (März et al., 2010a).

#### Neuroepithelial Progenitor Cells Are Maintained at the Adult Pallial Edge

Neuroepithelial (NE) cells are also present laterally and posteriorly at the junction of the pallium with the choroid plexus, a location corresponding to the dorsal midline (Figure 1E). These cells are ventricular and apico-basally polarized, express neither astroglial markers nor *her4*, and are proliferating. Their lack of astroglial markers and *her4* expression, and their cuboidal as opposed to radial shape, distinguish them from RG. “Negative” tracing of NE cells in the adult pallium, using as landmark a neighboring *her4.1:ERT2CreERT2* traced domain, suggests that these cells generate neurogenic RG in the postero-lateral pallial domain, as well as maintain themselves, acting as a local growth zone akin to the ones described in the adult optic tectum and retina (Dirian et al., 2014). Their exact lifespan and fate, however, remain to be studied in detail.

#### Highly Neurogenic Radial Glia Line the Subpallial Ventricle

RG cells also border the subpallial ventricle. They differ from pallial RG for their high levels of BLBP expression, their thick morphology, their higher proliferative potential, the interkinetic migration of their nuclei, and their generation of neurons that populate both the (subpallial) parenchyma and the olfactory bulb (Ganz et al., 2010; März et al., 2010a). Progenitors fated to the OB follow a longitudinal anterior-ward migration, akin to the rostral migratory stream of rodents, although glial corridors have not been observed (Kishimoto et al., 2011).

### Embryonic Origin of Adult Pallial Radial Glia: Heterogeneity, Functional Impact, and Comparison With NSC-Generating Lineages in Rodents

**FIGURE 2:**
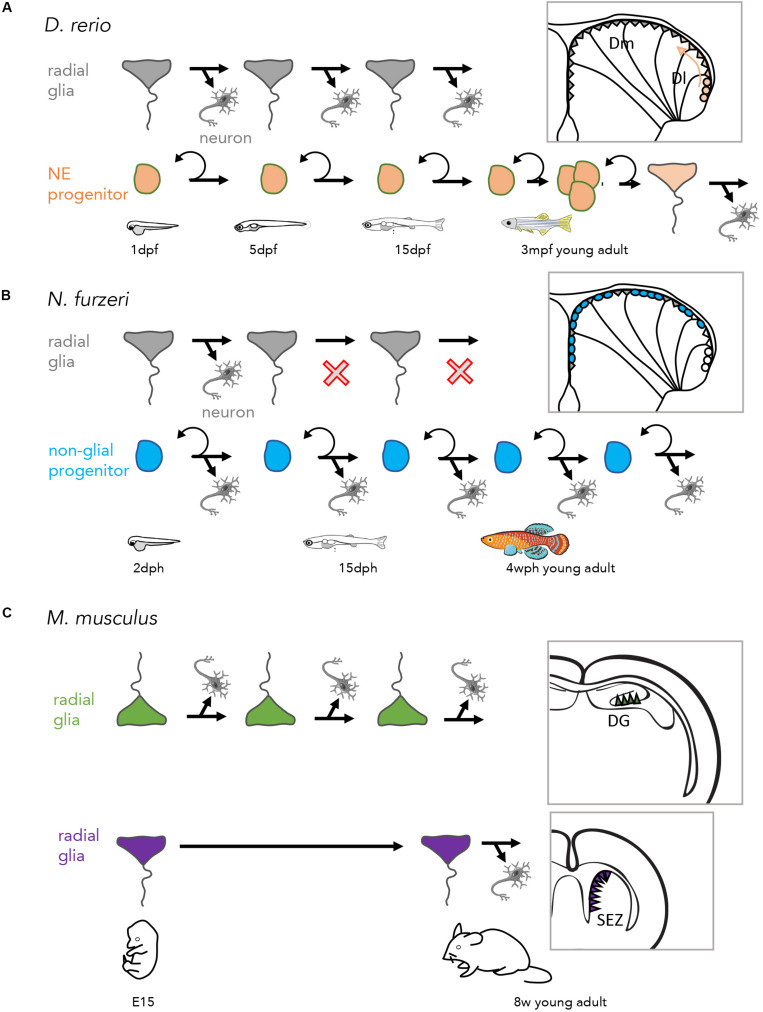
Lineages at the origin of adult neurogenic progenitors in the vertebrate pallium. **(A)** Lineages in zebrafish, generating adult RG from embryonic RG (top) and NE progenitors (bottom). **(B)** Lineages in the killifish, where neurogenesis in adults is ensured by a long-lasting non-glial embryonic lineage (blue) dph: days post-hatching, wph: weeks post-hatching. **(C)** Lineages in mouse, where distinct modes of NSC production are described in the DG (top) and SEZ (bottom) (Dirian et al., 2014; Furutachi et al., 2015; Song et al., 2018; Berg et al., 2019; Coolen et al., 2020).

Cre-lox lineage tracing indicates that pallial RG of the dorsal, medial and anterior pallial territories originate from embryonic RG that border the telencephalic ventricle at 1 day-post-fertilization (dpf) (Dirian et al., 2014). These embryonic RG, like their adult counterparts, express the Her transcription factor Her4. In contrast, as discussed above, adult RG of the postero-lateral pallium originate from the NE progenitor pool maintained at the pallial edge, itself deriving from dorsal NE progenitors located at the tel-diencephalic junction at 1 dpf (Dirian et al., 2014). NE progenitors express typical Her factors found at neural tube boundaries, such as Her6 and Her9, and *her9* expression is maintained into adulthood (Figure 2A). The two pallial RG populations remain separated by a cryptic boundary, and differ in a number of ways: posterior RG have a higher proliferation rate, higher expression of *blbp* and lower expression of GS (these three features possibly being related with their relatively “younger” age). Further, this population can be replenished from the NE pool if RG are depleted at larval stages.

Teleost fish encompass over 26,000 species across a large variety of habitats, and display a number of adaptations including in the morphology, growth rates or sizes of their pallium. As a response to its ephemeral habitat, *N. furzeri* follows an explosive development to its adult size (Blažek et al., 2013), including accelerated pallial growth and neurogenesis. Recent work demonstrates that this is not due to the enhanced efficiency of existing lineages, but rather to the long-term persistence until adulthood of a highly neurogenic embryonic lineage (Figure 2B; Coolen et al., 2020). This study, which points to the variety of neurogenic adaptations in the adult vertebrate brain, illustrates the power of fish models to uncover the different natural strategies that can be used to amplify neurogenesis.

Finally, in addition to their embryonic origin, a potential determinant of NSC properties is the duration of their neurogenic activity. Genetic tracing and birth dating experiments indicate that most, likely all, RG of the dorso-medial and anterior pallial domain originate from a constitutively neurogenic lineage, i.e., generating neurons without interruption from embryo to adult (Figure 2A; Dirian et al., 2014; Furlan et al., 2017). This was later shown to be also the case for NSCs of the adult mouse DG (Song et al., 2018; Berg et al., 2019). In apparent contrast, NSCs of the adult mouse SEZ were shown to derive from cells entering quiescence at mid-embryonic stages, hence pausing prior to being remobilized in adults (Figure 2C; Furutachi et al., 2015). It is possible, however, that quiescence instatement in the SEZ is more gradual and that an asynchrony exists in the control of quiescence entry and neurogenic activity among SEZ NSCs, reconciling the different models. Finally, it remains to be formally demonstrated whether the NE progenitors located at the pallial edge, and the young RG that they progressively generate *de novo* in the adult pallium, have an equivalent in rodents.

### Adult Neurogenic Lineages in the Zebrafish Pallium Are Devoid of Amplification and Drive Neuronal Addition

#### Different Amplification Strategies in Teleosts and Rodents

**FIGURE 3:**
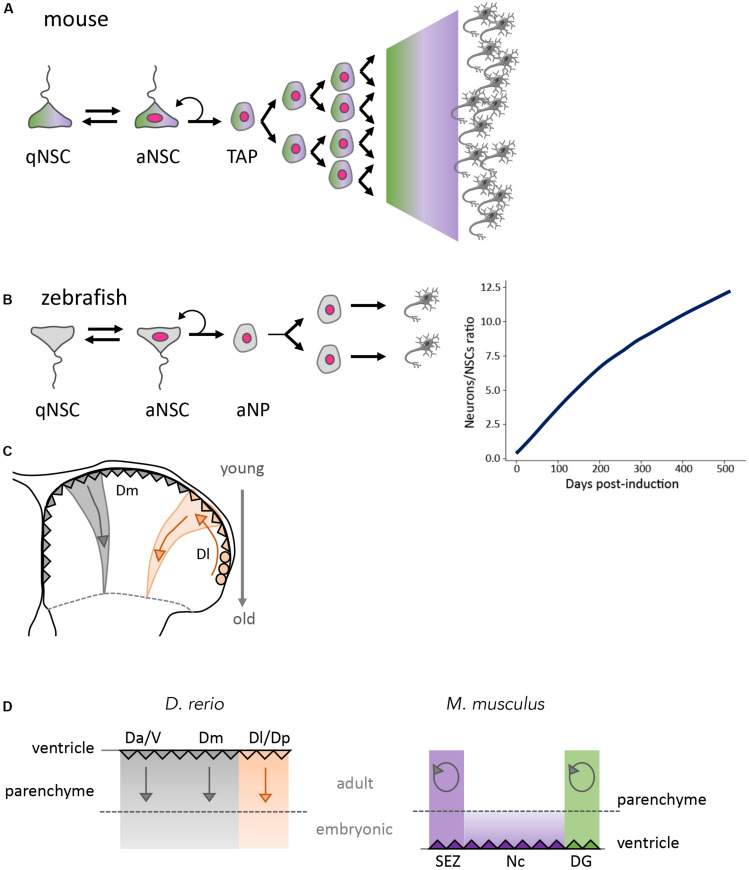
Global outputs of adult neurogenesis in zebrafish and mouse. **(A)** Scheme of a typical neurogenesis lineage in adult mouse. Upon quiescence exit, NSCs generate neurons via TAPs. TAPs have variable amplification capacity, high in the SEZ, lower in the DG. Green and purple shades are meant to represent shared cells and attributes between the SEZ and DG (color code in Figure 2C, with proliferating cells indicated with a pink nucleus). **(B)** Scheme of a typical neurogenic lineage in the adult zebrafish pallium (left) and neuronal output (right). Neurons are generated via an intermediate progenitor (NP: neural progenitor) of limited amplification potential. Because adult-generated neurons persist, however, the number of neurons generated per NSC increases over time in genetically traced lineages from individual NSCs. **(C)** Spatio-temporal distribution of the neurogenesis output in the zebrafish pallium, from embryonic stages until adult life. Radial glia (triangles) generate neurons that stack in age-related order within the telencephalic parenchyma. Old neurons, at the pallial-subpallial boundary, were generated in the embryo and early larva. In the lateral pallium (orange), the same process operates but radial glia are generated during juvenile and adult stages from NE progenitors (circles). Arrows indicate the spatial organization of neurogenesis over time. **(D)** Compared output of neurogenesis in the pallium of zebrafish and mouse from embryo to adult, represented on schematic cross-sections where the dotted line separates neurons generated at embryonic versus post-embryonic stages. Neurogenesis is continuous and additive (straight arrows) in zebrafish in all pallial subdivisions (left panel). Neurogenesis stops at birth in the mouse neocortex, spatially isolating the two persisting neurogenic niches SEZ and SGZ. Neurogenesis in these niches is mostly used for neuron replacement (circular arrows) (right panel). Color code as in Figure 1 (Seri et al., 2004; Encinas et al., 2011; Rothenaigner et al., 2011; Lugert et al., 2012; Ponti et al., 2013; Furlan et al., 2017; Than-Trong et al., 2020). D, dorsal part of the telencephalon (pallium); Da, anterior part of D; Dm, medial part of D; Dl, lateral part of D; aNSC, activated neural stem cell; qNSC, quiescent neural stem cell; NP, neural progenitor; TAP, transit amplifying progenitor; V, ventral telencephalon (sub-pallium).

Downstream of NSCs, adult neurogenesis in mouse involves TAPs, i.e., non-stem neuronal progenitors of limited self-renewal. The amplification potential of TAPs greatly varies between the SEZ and DG: in average, a TAP would divide three to four times in the SEZ (Ponti et al., 2013), but once or twice in the DG (Seri et al., 2004; Encinas et al., 2011; Lugert et al., 2012; Figure 3D). TAP-like progenitors are also present in the developing mouse cortex, notably as basal progenitors expressing the transcription factor Tbr2. These basal progenitors originate from RG and generate cortical neurons following 1 or 2 divisions (Hevner, 2019). Tbr2 expression is also found in the adult SEZ in amplifying progenitors generated from the TAPs (Lugert et al., 2012; Nelson et al., 2020). *tbr2* (*eomesa*) expression in the adult zebrafish pallium is largely regional and has not been directly associated with NPs (Ganz et al., 2015). The lineage amplification by pallial NPs is minimal, with at most one or two divisions, akin to TAPs of the DG (Figure 3C; Rothenaigner et al., 2011; Furlan et al., 2017). Hence, in the zebrafish pallium, extensive neuronal production is ensured by the continuous neurogenic activity of RG (notably in the cortical area, where neurogenesis is shut-down after birth in mammals) and the *de novo* addition of neurogenic RG into the system. The latter occurs through the activity of NE progenitors at the pallial edge, and currently unidentified “source cells” disseminated at the pallial ventricle (see below) (Than-Trong et al., 2020).

#### Adult Neurogenesis in Zebrafish Is Additive

Adult neurogenesis in mouse is globally understood to drive neuronal replacement, following the selective maintenance of a subset of adult-born neurons in the functional circuitry -while most adult-generated neurons would be eliminated (Figure 3B). Some publications, however, report neuronal addition, both in the DG (Bayer, 1985; Dranovsky et al., 2011) and OB (Platel et al., 2019). The output of pallial neurogenesis in zebrafish primarily drives neuronal addition. No cell death was observed, and the pallial parenchyma (as well as the OB) increases its neuronal population during adult life and grows (Than-Trong et al., 2020). Genetic birth dating and lineage tracing experiments showed that newborn neurons delaminate from the ventricular zone and stack into the parenchyma in age-related layers until adulthood (Furlan et al., 2017; Figure 3C). Because there is no extensive neuronal migration, and little or no death, this process results in an adult pallium where superficial structures are composed of young (late-born) neurons and central structures of old (early-born) neurons, still including neurons born at embryonic and early juvenile stages. This also applies to the lateral pallium, with in addition a lateral to medial gradient in RG age (Figures 3A,B; Furlan et al., 2017).

To date, the identity of adult-born pallial neurons, as well as their projection pattern and function, remain largely unknown in zebrafish. Like in the mouse, some adult-born neurons in the zebrafish OB are TH-positive (Adolf et al., 2006). In the pallial parenchyma proper, only candidate markers have been tested to date to characterize RG-derived neurons, including some transcription factors and neurotransmitters (identifying for example GABA-ergic and glutamatergic neurons) (von Trotha et al., 2014; Furlan et al., 2017). A neuron atlas was recently generated from the zebrafish telencephalon at 21 dpf using scRNAseq (Raj et al., 2018), and such a description is long awaited in adult, to permit both functional studies -still conducted currently through laborious screening to associate molecularly defined subpopulation with a given function (Lal et al., 2018)- and information on how NSCs generate different neuronal types. In the developing mammalian cerebral cortex and the Drosophila optic lobe, columnar organization is generated through sequential expression of specific transcription factors (Mattar et al., 2015; Doe, 2017). The zebrafish pallium is also built through a sequential stacking process, but in contrast to the mouse, the “migration-free death-free” neurogenesis process of the adult zebrafish pallium makes it possible to readily identify neurons born at adulthood by their (superficial) position (Furlan et al., 2017). This will then make it straightforward to attribute them with molecular signatures. Determining whether neurons at different depths have different identities and when they are generated would therefore represent an important step to know whether there is a temporal heterogeneity in NSCs and how it might be encoded. Moreover, since the same NSCs remain active in an adult brain which keeps on growing, one important question would then be whether NSCs maintain a similar level of plasticity throughout life, either physiologically or in a regenerative context. Finally, identifying neural subpopulations in the pallium could also reveal depth-independent areal heterogeneities, perhaps to be correlated with areal differences in NSC potential.

#### Pallial Neurogenesis in Zebrafish Is the Output of a Proliferative Hierarchy Involving Functionally Specialized NSC Sub-Pools

**FIGURE 4:**
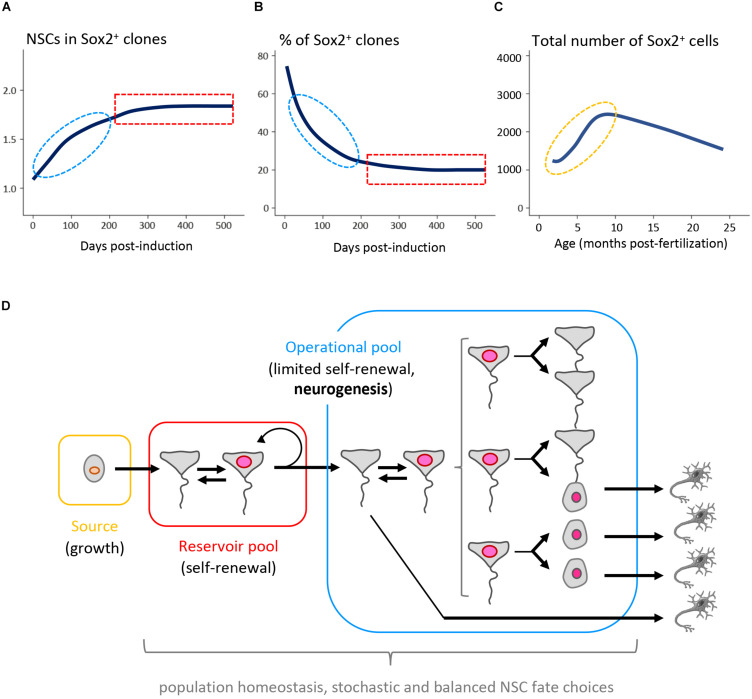
Long-term NSC and neurogenesis dynamic in the adult zebrafish pallium. **(A,B)** Genetic clonal analysis driven by the *her4:ERT2CreERT2* transgene with chase time over 500 days. The number of NSCs per clone containing at least one Sox2+ cell **(A)** and the proportion of clones containing at least one Sox2+ cell **(B)** display a bi-phasic dynamics at long term. At early time points after induction, neutral drift is observed -red-. At later time points, a behavior characteristic of single cell-based self-renewal appears -blue-. These two dynamics reflect the behavior of two embedded populations (operational and reservoir, respectively). **(C)** Total number of Sox2+ cells in the adult Dm between 3 and 25 months post-fertilization (mpf). The Sox2+ population increases in size in the young adult (3–8 mpf), reflecting the NSC-generating activity of a “source” population (orange). **(D)** Schematic of the proliferative hierarchy of NSC sub-populations sustaining overall NSC maintenance in Dm. Color code as in **(A–C)** (Than-Trong et al., 2020).

The zebrafish adult pallium is particularly amenable to NSC fate studies for several reasons: (i) its superficial location permits intravital imaging hence the direct tracing, during several weeks, of NSC fate in the absence of biased genetic tools and under non-invasive conditions (Barbosa et al., 2015b; Dray et al., 2015), (ii) its small size permits analyzing clones in whole-mount preparations, avoiding the risk of losing cells that occurs when studying brain sections, and (iii) the absence of cell death and migrations makes it easier to quantify clones in their entirety (Than-Trong et al., 2020). We made use of these attributes, and of broad promoters such as *her4* and *gfap* that encompass the largest progenitor population, to determine the dynamics of NSC fates in the adult pallium between 3 and 18 mpf (Than-Trong et al., 2020). The combination of intravital imaging, long-term clonal genetic tracing (Figures 4A–C) and biophysical modeling revealed that NSC population dynamics is compatible with an organization in 3 hierarchically-organized sub-populations, each endowed with a specific function: NSC population growth (“source pool”), self-renewal (“reservoir pool”), and neurogenic activity (“operational pool”) (Figure 4D). The “source” population accounting for growth remains poorly defined. In contrast, division modes and transition rates could be inferred for the reservoir and operation sub-populations, highlighting the heterogeneities of NSC properties and, within the operational pool, their stochastic fate choices.

In contrast to this unifying conclusion, the results of a large number of careful clonal studies in mouse diverge, documenting NSC loss, maintenance or even gain, in the SEZ and/or DG (Lugert et al., 2010; Bonaguidi et al., 2011; Dranovsky et al., 2011; Encinas et al., 2011; Fuentealba et al., 2012; Calzolari et al., 2015; Urbán et al., 2016; Basak et al., 2018; Bast et al., 2018; Obernier et al., 2018; Pilz et al., 2018; Berg et al., 2019). The zebrafish data suggest that these discrepant results could be interpreted by the targeting of distinct NSC sub-populations, although a unifying model in mouse remains to be established.

## Neural Stem Cell Quiescence and Its Impact on Neurogenesis

### Quiescence Is an Actively Maintained State Shared Between Zebrafish and Mouse Adult Neural Stem Cells

Quiescence is a prominent cell state in adult NSCs, as illustrated in both zebrafish and mice. It is therefore important to consider how it may affect NSC biology and neurogenesis output, likely in a similar way in these species. The quiescence phase of adult NSCs generally corresponds to the G0 state of the cell cycle. In Drosophila, NSCs can also undergo a G2 quiescence phase at late embryonic stages (Otsuki and Brand, 2018), and the existence of a long G2 phase has been suggested in NE progenitors of the medaka optic tectum at post-embryonic stages, based on the expression of G2-M arrest genes (Dambroise et al., 2017). G2 quiescence, however, remains to be demonstrated in vertebrate adult brains.

**FIGURE 5:**
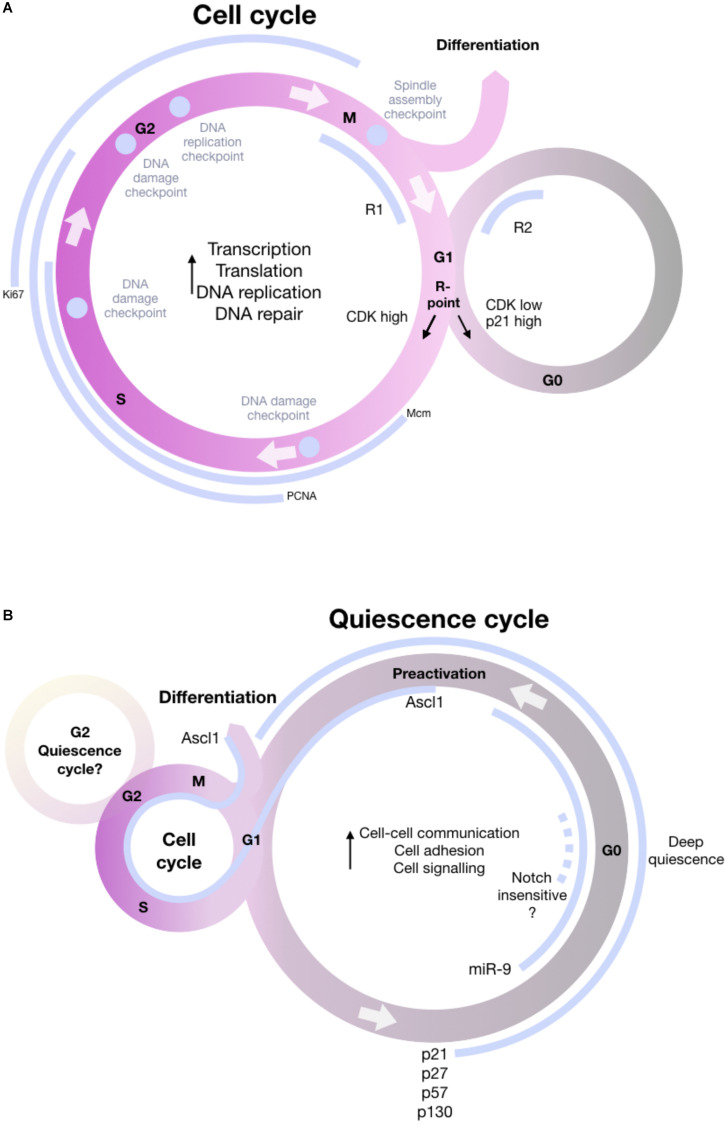
Schematic of the cell cycle including the most important information about the decision to enter quiescence, remain in cycle or differentiate. **(A)** General cell cycle knowledge, illustrating phases G1, S, G2, and M and the most important checkpoints (purple). During the cell cycle, proteins involved in transcription, translation, DNA replication and DNA repair are upregulated. The schematic includes proliferation markers MCM, PCNA, and Ki67 (gray) that are expressed in different phases of the cell cycle and commonly used to define proliferating NSCs. During the cell cycle, cells can enter into the quiescence state in G1, the decisions for entry happening at a R-point in G1. After passing the R-point, cells are committed to fulfill another cell cycle. Another important check-point is the bifurcation point right after mitosis, a window in which cells are sensitive to mitogen signals that influence CDK2 (R1 and R2 window on the schematic). Cells with a normal level of CDK2 will keep cycling, whereas cells with low levels of CDK2 will enter a transient quiescence and will face a second restriction window at the end of G1, controlled by the CDK inhibitor p21. Only cells that built up enough CDK will be able to bypass quiescence and eventually re-enter quiescence. **(B)** NSC-specific quiescence cycle. Quiescence can be entered in G1, or G2 (this remains to be shown for vertebrates). During quiescence, genes involved in cell-cell communication, cell adhesion and cell signaling are upregulated, stressing that quiescence is an actively maintained state. Some data (e.g., the dynamics of miR-9 expression) suggest that quiescence can be seen as a cycle, but alternative models exist. Quiescent cells express p21, p27, p57, and p130. Quiescence is heterogeneous, and deeper and shallower sub-states exist. miR-9 is nuclear in deeply quiescent cells. Some NSCs that are insensitive to Notch blockade can also be interpreted as deeply quiescent. A “pro-activated” state precedes activation proper. In this state, NSCs express ascl1, which will also be maintained during activation and differentiation (Pardee, 1974; Alunni et al., 2013; Spencer et al., 2013; Andersen et al., 2014; Katz et al., 2016).

Practically, quiescent NSCs are negatively defined by the absence of proliferation markers. Until now, a positive core signature for quiescent NSCs has not been defined, although RNASeq data in both mouse and zebrafish brought deeper understanding of the molecular players of NSC quiescence: generally, pathways involved in transcription, translation, DNA replication and DNA repair, and cell cycle progression, are downregulated (Codega et al., 2014; Dulken et al., 2017), while cell-cell communication (Shin et al., 2015; Basak et al., 2018), cell adhesion (Codega et al., 2014; Shin et al., 2015), cell signaling and lipid metabolism (Llorens-Bobadilla et al., 2015; Than-Trong et al., 2018) are upregulated (Figure 5).

Quiescence is classically linked with the maintenance of stem cell properties (stemness, i.e., self-renewal and differentiation potential, see Box 1). This link is, however, not obligatory, as illustrated in the gut and skin, where adult stem cells proliferate continuously while staying in homeostasis. In the brain, however, quiescence is believed necessary for stemness -hence neurogenesis potential-, avoiding DNA, protein or mitochondria damage that could lead to senescence or malignant transformations. But this has been difficult to demonstrate, both in mouse and zebrafish, in particular because testing for stemness requires functional assays where NSCs will divide, and because many quiescence control factors have pleiotropic effects and in particular are actors of the neurogenesis cascade itself (see below). Nevertheless, several studies to some extent disentangled the two properties. For example, in adult mouse, physical exercise leads to increased SGZ NSC proliferation, but is not followed by exhaustion of the NSC pool (Van Praag et al., 1999; Wu et al., 2008). In the adult zebrafish pallium, bulk RNAseq profiling of quiescent versus activated NSCs or in the presence or absence of Notch3 activity showed that Notch3 promotes quiescence and stemness in part via distinct molecular cascades (Than-Trong et al., 2018). While the transcription factor Hey1 mediates Notch3 activity on stemness, the candidate Notch3 effectors controlling quiescence remain to be experimentally validated. In mouse, the direct effect of Notch on stemness remains to be unraveled, as well as whether Hey1 is a target of Notch and could potentially control stemness. In the mouse SGZ, Notch2 drives expression of the transcription factor-encoding gene *Id4*. However, unlike the depletion of Notch2, the depletion of Id4 induces NSC activation but does not promote neuronal differentiation (Zhang et al., 2019). Thus, in mouse, NSC quiescence and stemness could also be molecularly uncoupled downstream of Notch2, Id4 controlling only its quiescence-promoting effect.

### Quiescence Instatement, Length, and Depth in Adult Neural Stem Cells: Variable Geometry Parameters?

#### Quiescence Length Remains to Be Measured With Precision

Through genetic lineage tracings and live imaging in zebrafish and mouse, we know now that NSCs can re-enter quiescence after activation (Berg et al., 2010; März et al., 2010a; Bonaguidi et al., 2011; Dray et al., 2015; Pilz et al., 2018; Than-Trong et al., 2020). It remains, however, unclear, and debated, whether NSCs keep the same properties (fate, quiescence length…) upon division (Bonaguidi et al., 2011; Urbán et al., 2016; Than-Trong et al., 2020). Quiescence length, as well the duration of cell cycle phases, also remain to be precisely defined in NSCs, and key studies on these issues are summarized in Table 2. Overall, S-phase can last between 4 and 8 h (Encinas et al., 2011; Ponti et al., 2013), and the complete adult NSC cell cycle will take 10–35 h (Encinas et al., 2011; Ponti et al., 2013; Roccio et al., 2013). The time between 2 divisions can lie between 14 and 36 days, as observed by live imaging in the SGZ, but the upper limits of quiescence were not explored (Pilz et al., 2018). In the zebrafish adult pallium, mathematical models predict average quiescence times reaching 143 days, which is yet to be confirmed experimentally (Than-Trong et al., 2020). It is likely that the zebrafish pallium will be highly instrumental to fill these gaps, as the superficial location of the pallial progenitor zone (contrasting with the deep location of mammalian NSCs) permits long-term intravital imaging.

**TABLE 2:** Estimated lengths of cell cycle phases and quiescence in adult NSCs of the zebrafish and mouse telencephalon.

Cell cycle phase	Length	Model	Method	References
G1	0–35 h, most of the time: 6–15 h	Mouse NSC cell culture	*Hes5:FUCCI* line	Roccio et al., 2013
S/G2/M	4–12 h, most of the time: between 4 and 9 h	Mouse NSC cell culture	*Hes5:FUCCI* line	Roccio et al., 2013
Complete cell cycle	17 h	Mouse NSCs in the SEZ	Thymidine analog	Ponti et al., 2013
S-phase	4.5	Mouse NSCs in the SEZ	Thymidine analog	Ponti et al., 2013
S-phase	ANP (amplifying neural progenitors, higher level of proliferation): 12.2 ± 1.1 QNP (quiescent neural progenitors, low level of proliferation): 7.8 ± 0.7	Mouse NSCs in the SGZ	Thymidine analogs	Encinas et al., 2011
Cell cycle	28–35 h	Mouse NSCs in the SGZ	Thymidine analogs	Encinas et al., 2011
G0	20 ± 4 days	Mouse NSCs in SEZ	Genetic tracing based on Wnt-target *Troy:GFP.* In the model, qNSCs become activated at constant low rate, and aNSC go to quiescence at constant rate.	Basak et al., 2018
G1 G0 transition	5 ± 2 days	Mouse NSCs in SEZ	Genetic tracing based on Wnt-target *Troy:GFP.* In the model, qNSCs become activated at constant low rate, and aNSC go to quiescence at constant rate.	Basak et al., 2018
Division rate	5 ± 2 h	Mouse NSCs in SEZ	Genetic tracing based on Wnt-target *Troy:GFP.* In the model, qNSCs become activated at constant low rate, and aNSC go to quiescence at constant rate.	Basak et al., 2018
Non-proliferating		Mouse NSC in the SGZ	Live imaging with inducible *Ascl1:tdTomato* line	Pilz et al., 2018
G0	24.4 and 143 days	Zebrafish pallium	Genetic tracing based line *Tg(her4:RFP).* In the mathematical model, qNSCs become activated at 2 different rates.	Than-Trong et al., 2020

#### Quiescence Instatement Is Progressive With a Schedule That May Differ Between Niches and Species

Progenitors in the SGZ produce granule neurons during embryonic and postnatal stages and enter quiescence postnatally. Then, they acquire their radial morphology and organize in the SGZ (Li et al., 2013; Berg et al., 2019). In contrast, in the SEZ, stem cells with quiescence characteristics were identified at embryonic stages by H2B-mediated lineage tracing (Furutachi et al., 2015). These cells would slow down their cell cycle at E13.5, then remain quiescent to re-activate at adult stages (Fuentealba et al., 2012). As mentioned earlier, these differences between the SEZ and SGZ may be apparent and due to tracing some cells only, or due to using indirect measurements. For example, H2B-tracing is based on differential dilution, and a positive read-out necessitates a minimal quiescence length. In zebrafish, pallial neural progenitors start entering quiescence at 5 dpf (Alunni et al., 2013), and the average duration of quiescence -as inferred from the decreasing proportion of PCNA-positive cells within the population- gradually increases until adulthood (Dirian et al., 2014; Katz et al., 2016). It remains unclear whether the data above can directly be compared, as they use different methods with their inherent limitations. Likewise, measures based on the lack of PCNA protein will not distinguish cells in early G1 phase (PCNA transcription and protein stability being low prior to the G1-S transition) (Chang et al., 1990) from cells in G0. Progressive quiescence instatement, concluded from the increasing duration of a PCNA-negative state, may therefore be concluded for cells that in fact progressively lengthen early G1. Overall, it remains urgent for the field to positively label G0.

#### NSC Quiescence Is a Heterogeneous State

Several analyses support the idea that G0 quiescence is heterogeneous. Some studies suggest different types of quiescence (mainly short versus long-term) depending on the cell and its history (Urbán et al., 2016). Additionally, quiescence can consist of sub-states, defined as transient phases, arguably harboring specific molecular or cellular signatures and properties, that cells transit through during their quiescence phase. Zebrafish adult pallial NSCs were instrumental to experimentally exemplify potential quiescence sub-states. For example, pharmacological blockade of Notch signaling in zebrafish, which globally leads to NSC quiescence exit (see below), revealed different lag phases to re-enter cycling, and approximately 5% of quiescent NSCs did not respond to the blockade (Alunni et al., 2013). Convincingly, a subset of quiescent NSCs express microRNA-9 (miR-9), and BrdU chase experiments suggest that the miR-9-positive state is a transient phase in a quiescence cycle and may reflect deep quiescence (Katz et al., 2016). Indeed BrdU incorporated during the S phase of dividing NSCs becomes associated with miR-9 staining only after long chase, showing that miR-9 is expressed in now deeply quiescent NSCs but that were previously dividing.

scRNAseq and expression analyses conducted in mouse also suggest the existence of a distinct quiescent sub-state close to activation, as was proposed for muscle satellite cells. The first study reporting such heterogeneity in the mouse SEZ identified three non-dividing NSC clusters (Llorens-Bobadilla et al., 2015): a dormant cluster in the deepest state of quiescence, a second cluster containing cells expressing markers related to activation but that do not divide, and a third cluster that falls in between on the spectrum between quiescence and activation. The same group reported the same subpopulation structure in a new study and using a different technology, suggesting that these cells can be reliably and reproducibly grouped into distinct clusters (Kalamakis et al., 2019). Recently a separate group reported the most extensive scRNAseq conducted on NSCs so far (Mizrak et al., 2019), in which they captured close to 40k SEZ astrocytic cells. They identified several independent clusters that also matched distinct regions along the SEZ which differ in proliferation rate. This can be explained if NSCs along the lateral ventricles rest in different depths of quiescence. An important limitation to these experiments in the SEZ, however, is the difficulty to distinguish between astrocytes and *bona fide* NSCs (Dulken et al., 2017). In the dentate gyrus, astrocytes and RGL-cells formed distinct clusters (Hochgerner et al., 2018). However so far only low numbers of stem cells were captured in scRNAseq experiments conducted on the hippocampus, which prevents proper analysis of their intrinsic heterogeneity. Two reports were recently published in zebrafish, based on NSCs isolated from *her4.1*-driven transgenes (Cosacak et al., 2019; Lange et al., 2020). However these studies only captured small numbers of NSCs (609 and 76, respectively). One of them conclusively shows the existence of distinct NSC clusters in the pallium (Cosacak et al., 2019), but more extensive studies will be necessary to get a better idea of the level of heterogeneity as well as whether and how these subpopulations differ in quiescence depth.

### Control Mechanisms of Quiescence Are Highly Conserved Between Zebrafish and Rodents

**FIGURE 6:**
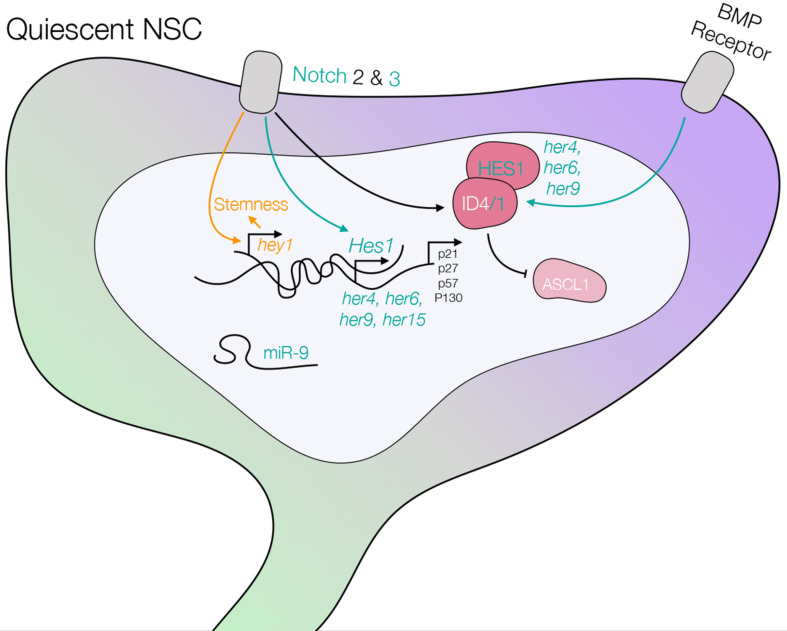
Schematic of a quiescent NSC including the pathways controlling quiescence, which are summarized in this review. The scheme highlights knowledge generated in mouse, and confirmed pathways in zebrafish are illustrated in green. Knowledge generated in zebrafish and later extended to mouse is shown in green as well. Knowledge generated in zebrafish and still to be confirmed in mouse is depicted in yellow. Differences (Notch2 is not expressed in qNSCs in zebrafish), or data that need consolidation in zebrafish (Ascl1 expression and its regulation by ID and Hes1, BMP receptor), are shown in black. See text and Table 3 for references.

Control mechanisms of NSC quiescence in zebrafish and rodents appear similar, yet many mechanisms that were identified in rodents remain to be studied in zebrafish and vice versa. Conditional functional studies in the adult zebrafish remain technically challenging, especially when genetics-based, and this is still slowing down the field. We will focus here only on the control mechanisms that have been studied in the zebrafish pallium and compare them to data in mouse (Figure 6) (but see Table 3). It is to note that, while these studies convincingly implicate various factors in quiescence control, they do not resolve their function in controlling quiescence entry, maintenance, exit, or transition through the different sub-states discussed above.

**TABLE 3:** Quiescence promoting factors.

Quiescence promoting factor	Model	Method	References
Notch (in general)	Mouse SGZ	Conditional knockout of *RBPJk* in *Glast-*expressing NSCs enhances neurogenesis (quiescence exit) and leads to NSC depletion 2 months after induction	Ehm et al., 2010
	Mouse SEZ	Conditional knockout of *RBPJk* in *Nes*-expressing NSCs enhances neurogenesis (quiescence exit) and leads to NSC depletion 3 months later	Imayoshi et al., 2010
Notch3	Adult zebrafish pallium	Pharmacological blockade of gamma-secretase (Notch signaling pathway), *notch3* mutant and *notch3* MO show that Notch3 maintains quiescence	Alunni et al., 2013
	Mouse SEZ	*Notch3-*null mice and knockdown in adult with lentiviral expressing shRNA targeting *Notch3* drive quiescence exit of NSCs, especially in the lateral and ventral wall	Kawai et al., 2017
Notch2	Mouse SEZ	Conditional knockout of *Notch2* in *Hes5*-expressing NSCs and short-term lineage tracing of Notch2-expressing cells shows that Notch2 maintains NSCs in quiescence, as loss of function leads to quiescence exit, increased neurogenesis and accelerated NSC exhaustion	Engler et al., 2018
	Mouse SGZ	Conditional knockout of *Notch2* in *Hes5*-expressing NSCs leads to proliferation of NSCs and increased neurogenesis	Zhang et al., 2019
Dll1	Mouse SEZ	Conditional knockout of *Dll1* in *Nes*-expressing NSCs leads to quiescence exit (feedback to activate Notch in quiescent cells is not ensured anymore)	Kawaguchi et al., 2013
Jagged1	Mouse SGZ	Conditional knockout of *Jagged1* in *Nes*-expressing NSCs leads to quiescence exit, implying that the interaction between Jagged1 and Notch is important for NSC quiescence and maintenance	Lavado and Oliver, 2014
Fezf2	Adult zebrafish pallium	Vivo-morpholino against *fezf2* (short-term knock-down) leads to quiescence exit and increased proliferation	Berberoglu et al., 2014
Bone morphogenic proteins	Mouse SEZ	BMP7 overexpression (virus-mediated) and Noggin expression (through protein purification or virus-mediated) show that expression of BMP maintains quiescence in type B cells/NSCs and therefore inhibits neurogenesis. It promotes the survival of type A progenitors	Lim et al., 2000
	Mouse SGZ	Blocking BMP through Noggin leads to reactivation and expansion of the NSC pool, suggesting that BMP is involved in quiescence control	Bonaguidi et al., 2008
	Mouse SGZ	Intracerebral infusion of Noggin, lentivirus-mediated ablation of *BMPR-1A* and conditional knockout of *Smad4* in *Glast*-expressing NSCs lead to quiescence exit, increased proliferation and exhaustion	Mira et al., 2010
	NSCs derived from ESCs	NSC culture can be pushed to quiescence by adding BMP4 in 24 h	Martynoga et al., 2013
Inhibitors of DNA binding	Mouse SGZ	Conditional knockout of *Id4* in *Glast*-expressing NSCs leads to increased ASCL1 expression and reactivation of previously quiescent NSCs	Blomfield et al., 2019
	Mouse SGZ	Conditional knockout of *Id4* in *Gfap*-expressing cells using *adeno-gfap::Cre* viruses leads to NSC activation and cell-cycle entry without inducing neurogenesis	Zhang et al., 2019
NFIX	NSC culture derived from Mouse ESCs	Knockdown *in vitro* leads to impaired quiescence	Martynoga et al., 2013
	Mouse	*NFIX-/-* knockout: lethal at 3 weeks. Proportion of cycling NSCs is increased in the mutant.	Martynoga et al., 2013
Forkhead box O3	Mouse SEZ and SGZ	Conditional knockout of FOXO1,3,4 in *Gfap*-expressing cells: decline of NSC pool and neurogenesis.	Paik et al., 2009
	Neurospheres from NSC culture	Neurospheres from *FOXO3-/-* versus *FOXO3+/+*, genome-wide microarray analysis: FoxO3 induces a program to preserve quiescence	Renault et al., 2009
	Mouse SEZ and SGZ	*FOXO3-/-:* reduced number of NSCs *in vivo* (NSCs got activated and lost)	Renault et al., 2009
	Adult mouse primary NSC culture	ChIP: FOXO3 binds proneural genes that are also targeted by Ascl1 possibly as a competitor to repress their expression and maintain NSC identity	Webb et al., 2013
	Mouse SGZ	Conditional knockout of *FoxO1,3,4* in G*last*-expressing NSCs: leads to quiescence exit, increased proliferation followed by loss of NSC number	Schäffner et al., 2018
	Mouse SEZ	*FoxO3-/-* knockout results in quiescence exit and increased neurogenesis	Webb et al., 2013
miR-9	Zebrafish pallium	Vivo-MO targeting mature miR-9 leads to reactivation of previously quiescent mir-9+ NSCs.	Katz et al., 2016
Gaba	Mouse postnatal SEZ, acute slices	GABA_*A*_-R-antagonist bicuculline leads to increase of proliferation in GFAP+ cells	Liu et al., 2005
	SGZ adult	Clonal analysis after cKO of gamma_2_-subunit-containing GABA_*A*_ receptor in *Nes-*expressing NSCs – > quiescence exit and symmetrical self-renewal	Song et al., 2012
	SGZ adult	Pharmacological inhibition – > increase of NSC proliferation Genetic deletion of GABA_*B1*_ (homozygous mutant) – > increase of NSC proliferation (Sox2+ cells) and differentiation to neuroblasts. Later loss of progenitors and increased neurogenesis	Giachino and Taylor, 2014

#### Notch Signaling Is a Key Quiescence-Promoting Pathway

One of the most prominent NSC quiescence-promoting pathway is Notch signaling, as first demonstrated in the adult zebrafish pallium (Chapouton et al., 2010), and later confirmed to be conserved by numerous studies in mice. In zebrafish and mice, Notch is highly expressed in NSCs. Whereas *notch3* is strongly upregulated in quiescent cells, *notch1* (*notch1b* in zebrafish) is strongly expressed in activated NSCs (Aguirre et al., 2010; Chapouton et al., 2010; Basak et al., 2012; Alunni et al., 2013; de Oliveira-Carlos et al., 2013; Kawai et al., 2017; Zhang et al., 2019). *notch2* expression was not detected in the zebrafish brain, but is expressed in quiescent NSCs in mice (Basak et al., 2012; Kawai et al., 2017; Zhang et al., 2019). Blocking Notch signaling with a gamma-secretase inhibitor dissolved in fish water leads to activation of NSCs and expansion of the NSC pool by symmetric divisions (Chapouton et al., 2010; Alunni et al., 2013), and this was recapitulated by the selective blockade of Notch3 using morpholinos (Alunni et al., 2013). In mice, Notch inhibition, either at the level of the ligands or the effector RBPjK leads to exit of quiescence and exhaustion of the NSC pool, a phenotype also often understood to mean that NSC quiescence is crucial for stemness maintenance (Ehm et al., 2010; Imayoshi et al., 2010; Kawaguchi et al., 2013; Lavado and Oliver, 2014). In zebrafish, NSC exhaustion was not observed, but long-term Notch blockade at adult stage was not conducted beyond 7 days (Alunni et al., 2013). One transcription factor functionally interacting with Notch signaling is Fezf2 (Fez family Zinc Finger 2), which is expressed at high levels in quiescent NSCs in the zebrafish adult pallium and mouse SGZ (Berberoglu et al., 2014). In zebrafish, *fezf2* expression correlates with the nuclear localization of NICD and with high expression level of the Notch target *her4*, and is necessary for quiescence (Berberoglu et al., 2014).

#### Other Quiescence Promoting Pathways Are Highly Conserved Between Rodents and Zebrafish

Another important pathway for NSC quiescence is BMP (bone morphogenic protein) signaling. NSCs express components of the BMP pathway like Smads, BMPR I and II (Lim et al., 2000; Bonaguidi et al., 2008; Mira et al., 2010). Overexpressing BMP ligands leads to a decrease in NSC proliferation and differentiation, while overexpression of the BMP inhibitor Noggin leads to increased proliferation and neurogenesis in the SGZ, the SEZ and *in vitro* (Lim et al., 2000; Bonaguidi et al., 2008; Martynoga et al., 2013). Targets of the BMP pathway include ID transcription factors (“Inhibitor of DNA binding/differentiation”), which are also targeted by the Notch pathway. IDs are strongly expressed in zebrafish adult pallial NSCs, and recent work shows that BMP positively controls *id1* expression through conserved enhancers in the adult zebrafish brain (Zhang et al., 2020). In zebrafish, *id1* expression is specific of quiescent NSCs, and is necessary and sufficient for quiescence (Diotel et al., 2015; Rodriguez-Viales et al., 2015). In response to injury, *id1* is upregulated. It may play a role in maintaining the NSC pool through stabilizing its interactor proteins such as the Her factors Her4 or Her6, also expressed in adult pallial NSCs (Rodriguez-Viales et al., 2015). In mouse NSCs, Id interacts with and stabilizes Hes1, the mammalian ortholog of zebrafish Her6. Hes1 represses the transcription factor Ascl1 (Bai et al., 2007), which itself normally promotes NSC activation (Andersen et al., 2014; Sueda et al., 2019). Id4 does not affect *Ascl1* transcription, but binds the normal Ascl1 stabilizing partner E47, leading to Ascl1 clearing (Blomfield et al., 2019).

Finally, the miR-9 quiescence-promoting factor initially identified in adult zebrafish pallial NSCs (see above) (Katz et al., 2016) is also conserved in mouse, as well as its striking sub-cellular localization: in both species, miR-9 is nuclear in NSCs transiting through a deep quiescence sub-state. Further, primary NSCs in culture derived from the SGZ and pushed toward quiescence through BMP relocalize miR-9 to the cell nucleus (Katz et al., 2016). The targets of miR-9 in quiescence control remain unknown.

Overall, a tentative quiescence cycle is presented in Figure 5B, indicating the transient-sub-states (miR-9-positive, Ascl1-positive, Notch-insensitive) that NSCs transit through.

#### Activating Factors Are Also Shared Between Rodents and Zebrafish

A key promoter of NSC activation, mentioned above, is Ascl1 (achete and scute homolog 1), which directly upregulates the expression of cell cycle genes (Castro et al., 2011; Andersen et al., 2014). Ascl1 is expressed in all activated NSCs and some neural progenitors in the mouse SEZ and SGZ. Conditional loss of function experiments showed that Ascl1-negative NSCs neither proliferate nor differentiate (Andersen et al., 2014). Ascl1 is transcribed in some quiescent NSCs (Blomfield et al., 2019; Zhang et al., 2019), but its expression and activity are repressed during quiescence by Id4 and the Notch target Hes1, which is expressed at high level with moderate oscillation amplitude (Sueda et al., 2019). How high Ascl1 expression levels become induced to drive NSC activation remains to be uncovered. Lower and oscillating levels of Hes1 expression preceding NSC activation can lead to Ascl1 oscillations, themselves driving NSC activation (Sueda et al., 2019). Then, following NSC division, the ubiquitin ligase HUWE1 degrades Ascl1 thus enabling the cell to re-enter quiescence (Urbán et al., 2016). In zebrafish, *ascl1a* is expressed in activated NSCs (Than-Trong et al., 2018), but its function remains to be studied. Further to this transcription factor, growth factors are also activating factors in NSCs. In the mouse brain, intracerebroventricular infusions of the fibroblast growth factor FGF2 lead to increased proliferation and neurogenesis (Rai et al., 2007). Accordingly, conditional knock-out of *FgfR1* in Nestin-expressing NSCs in the SGZ impairs proliferation and neurogenesis (Zhao et al., 2007). In the zebrafish brain, *fgfr1-4* are expressed in the dorsal telencephalon. Whereas heat shock-induced expression of dominant negative forms of FGFR1 does not alter NSC activation, the overexpression of FGF8a results in strong proliferation (Topp et al., 2008; Ganz et al., 2010). *fgf8a* expression is restricted to the ventral telencephalon, but *fgf8b*, strongly expressed in the pallium (Topp et al., 2008), may play the same role.

## Stemness-Related Neural Stem Cell Fate Choices

Decisions taken by NSCs along their life include whether to activate (or remain quiescent) but also whether to maintain (or lose) their stemness (Box 1). We will refer to “stemness-related NSC fate choices” the checkpoints when a NSC decides to remain stem or to commit toward expression of the genetic program reflective of another cell type.

### NSC Potency: Do NSC Fates Differ Between Zebrafish and Mouse?

In the SEZ, the differentiation potential of individual NSCs is limited to specific neuronal subtypes based on their regional localization (Merkle et al., 2007; Merkle et al., 2014; Chaker et al., 2016; Mizrak et al., 2019). However, fate mapping experiments confirmed that even if most NSCs produce neurons, few NSCs produce oligodendrocytes (Menn et al., 2006) or astrocytes (Sohn et al., 2015). Still, the capacity for a single NSC to produce the 3 lineages *in vivo* at adult stage remains unclear (but see Levison and Goldman, 1993 for the neonate). *In vitro*, clonal cultures of primary NSCs are able to generate neurons and oligodendrocytes (Menn et al., 2006) but continuous live-imaging of dividing NSCs revealed their commitment toward oligodendrogenic or neurogenic lineages only (Ortega et al., 2013). Also, ependymal cells were not described to originate from NSCs under physiological conditions (Spassky et al., 2005; Shah et al., 2018). NSCs of the SGZ most probably possess a heterogenous range of self-renewal and fate potential (Bonaguidi et al., 2012). Compared to the SEZ, clearer examples of multipotent NSCs were unraveled by careful analysis of lineage tracing outputs and notably of clones of 3–4 cells, showing that an individual NSC can self-renew and give rise to neurons and astrocytes (Bonaguidi et al., 2011; Encinas et al., 2011). While they do not give rise to oligodendrocytes physiologically, they can do so under conditions of demyelination or following the functional abrogation of inhibitory transcription factors (Nait-Oumesmar et al., 1999; Xing et al., 2014; Harris et al., 2018).

The situation in the adult zebrafish pallium is inherently different, as there are no “specialized” NSCs given that, as mentioned, RG cells also serve the function of parenchymal astrocytes. Thus, stemness maintenance includes the maintenance of astrocytic function (the reverse not being true, as stemness can be lost while astroglial characteristics are maintained; Than-Trong et al., 2018). At present, adult pallial NSCs are viewed as bipotent, able to self-renew and to generate neurons (Adolf et al., 2006; Grandel et al., 2006; Kroehne et al., 2011; Than-Trong et al., 2020). Little is known about neuronal subtypes in the dorsal pallium and it remains unexplored if specific NSC pools give rise to neuronal subtypes (like in the SEZ). The dorsal pallium is deprived of ependymal cells, but hosts an Olig2-positive population of oligodendrocytes. No clear lineage relationship has been made between oligodendrocytes and NSCs. The Olig2-positive population of cells is heterogeneous and located mostly in the parenchyma -although some cells can be found close to the ventricular surface-, comprising mature oligodendrocytes, slow proliferating oligodendrocyte progenitors (OPCs), proliferating OPCs, quiescent OPCs and radial glia-like cells (März et al., 2010b). These observations suggest that oligodendrocytes are produced within the parenchyma from OPCs. A recent publication based on scRNAseq argues that *her4.1*-positive NSCs express *olig2* at very low level, suggesting nascent NSC progeny differentiating toward OPCs (Lange et al., 2020). Likewise, pseudo-lineages inferred from scRNAseq in the mouse SEZ reveal a molecular connection between NSCs and oligodendrocytes (Mizrak et al., 2019). This hypothesis needs to be carefully tested with a lineage tracing approach.

These observations together suggest potential differences between the panel of fates endogenously taken by SEZ, SGZ, and pallial NSCs between mouse and zebrafish. Hence, stemness-related fate choices are complex and not limited to remaining or not stem, but may include the choice of a particular fate. These differences may reflect a different potential, or the presence of different contextual cues.

### Stemness-Related Fate Choices in Zebrafish Pallial NSCs Can Be Taken in the Quiescent or Activated States

#### Direct Neuronal Differentiation Is a Frequent Adult NSC Fate in Zebrafish

Both the quiescent and activated NSC states harbor potential windows where stemness can be maintained or altered. In the zebrafish adult pallium, the generation of neurons directly from quiescent NSCs has been suggested based on intravital imaging methods where NSCs were observed to differentiate after over 6–20 days without division (Barbosa et al., 2015b; Than-Trong et al., 2020). Thus, stemness needs to be actively promoted even during quiescence. Some effectors of NSC stemness maintenance have been identified in mice (Ars2 and Sox2) (Andreu-Agulló et al., 2009; Baser et al., 2019) and in zebrafish (Hey1) (Than-Trong et al., 2018). Interestingly, the depletion of their function in quiescent NSCs leads to a non-stem RG (GS^+^; Sox2^–^) fate suggesting that direct neuronal differentiation further requires active neuronal commitment cues.

#### The Mechanisms Driving NSC Fate Choices at Division Remain Poorly Understood

Adult zebrafish pallial NSCs can take several fates at division and generate two NSCs, one NSC and one aNP, or two aNPs. Models of clonal dynamics are compatible with stochastic decisions under physiological conditions (Than-Trong et al., 2020). Following a mechanical lesion, which leads to enhanced NSC recruitment for neuronal regeneration, a bias toward neurogenic consuming divisions (generating two aNPs) was observed (Barbosa et al., 2015b). In contrast, upon pharmacological Notch blockade, enhanced NSC recruitment is accompanied by a bias toward amplifying divisions (generating two aNSCs). It remains largely unknown how these decisions are taken.

**FIGURE 7:**
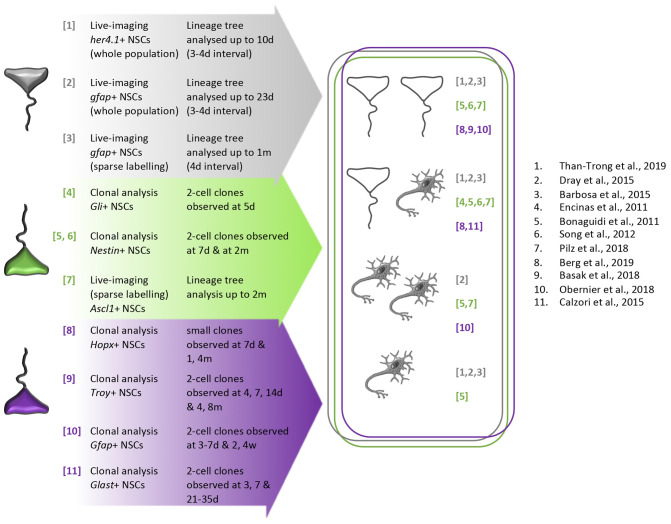
Schematic summary of division modes directly observed in adult mouse telencephalic neurogenic niches and in the zebrafish adult pallium *in vivo*. To evidence with certainty the existence of each division mode, we listed on the left part of the figure the clonal lineage tracing and live-imaging analyses only. For all the clonal analysis, we also only focused on 2–3-cell clones data at various time of induction/chase. Arrow depict the path leading individual NSC toward a cell fate decision (symmetrical self-renewing division, asymmetrical self-renewing division, symmetrical differentiating division or direct differentiation, illustrated on the right part of the figure). In the zebrafish pallium (gray NSCs), the mouse SEZ (purple NSCs), and the mouse SGZ (green NSCs), the three modes of division were evidenced. Direct neuronal differentiation was observed in the zebrafish adult pallium and mouse SEZ. In the SEZ, *Gfap+* and *Troy+* NSCs are able to symmetrically self-renew, symmetrically differentiate and asymmetrically divide whereas *Glast+* NSCs were only described to asymmetrically divide. In the SGZ, *Nestin+* and *Ascl1+* NSCs can symmetrically self-renew, symmetrically differentiate and asymmetrically divide although *Gli+* NSCs were only observed to asymmetrically divide and Hopx+ NScs to symmetrically self-renew and asymmetrically divide. Numbers refer to publications (see reference list).

Examples of all three division modes were also directly observed in mouse *in vivo* by clonal analysis of small clones (Bonaguidi et al., 2011; Encinas et al., 2011; Song et al., 2012; Calzolari et al., 2015; Basak et al., 2018; Obernier et al., 2018) or by live-imaging (Pilz et al., 2018). In both NSC niches, the choice for a given division mode seems to vary in part depending on the driver line used to follow NSCs, and the discrepancies may reflect experimental designs. Still, this observation would argue for the existence of NSC signatures highlighting specific modes of division (Figure 7).

Key studies in embryonic neural progenitors, including in zebrafish, pointed to several mechanisms controlling or biasing daughter cell fate at division. These notably include cell cycle dynamics such as the length of G1 or S phases (Calegari and Huttner, 2003; Huttner and Kosodo, 2005; Chen et al., 2015; Turrero García et al., 2016), asymmetrical inheritance of cellular components at division (Knoblich, 2008; Kressmann et al., 2015; Tozer et al., 2017; Lukaszewicz et al., 2019; Taverna and Huttner, 2019), and intra-lineage or niche-mediated bias in Notch signaling (Dong et al., 2012). Corresponding data in adult NSCs are sparse and were generally obtained *in vitro*, in mouse. For example, in cultured adult SGZ NSCs, G1 lengthening (through CDK4 inhibition) pushes NSCs toward differentiation (Roccio et al., 2013). Speculatively, basal process inheritance could be a fate determinant, as suggested by *ex vivo* SEZ cultures analyzed with live imaging (Obernier et al., 2018). Cultures of individual NSCs from the SEZ also showed asymmetric molecular segregations or activations. Specifically, the asymmetric segregation of the Dual-specificity tyrosine-phosphorylated and regulated kinase Dyrk1A at NSC division stabilizes EGFR and Notch signaling, biasing daughter cell fate (Ferron et al., 2010). Overexpressed Delta1-eGFP fusion protein also distributes asymmetrically upon NSC division, and marks the daughter cell fated to neuronal commitment (Kawaguchi et al., 2013). Finally, PEDF signaling from the niche can locally activate Notch in one NSC daughter (Ramírez-Castillejo et al., 2006; Andreu-Agulló et al., 2009). Parallels to these pioneer mechanistic works are currently lacking *in vivo*, and in zebrafish.

Stemness-related fate choices are key determinants of NSC population homeostasis, i.e., to the maintenance of a constant number of NSCs over time. Two mechanisms can in theory account for such homeostasis: invariant asymmetric cell fate, and “population asymmetry” (a combination of individual stochastic fate choices, balanced at the population level) (Simons and Clevers, 2011; Blanpain and Simons, 2013). In the mouse brain, NSC dynamics remains controversial (Table 4). As mentioned above, the privileged morphology of the zebrafish pallial germinal zone made it possible to combine complementary approaches and extract a unified model of adult NSC dynamics (Figure 4C). This resolved discrepancies between works describing an expansion (Rothenaigner et al., 2011) or a consumption (Barbosa et al., 2015b) of the NSC population. The current model (Than-Trong et al., 2020) includes both expansion and consumption but attributes these behaviors to distinct subpopulations of NSCs, and to stochastic fate choices. Further, it shows for the first time that both invariant asymmetric stem cell fate and population asymmetry can co-exist in an assembly of subpopulations hierarchically organized to account for NSC maintenance and physiological neuronal output.

**TABLE 4:** NSC population dynamics assessed by long-term lineage tracing and clonal analysis in mouse.

NSC niche	Methods	Chase time	Self-renewal capacity	Population dynamics	Population dynamic mechanism	References
SGZ	*Nestin:CreERT2* lineage tracing + BrdU labeling	Up to 30 days	Limited	Progressive consumption of the NSC pool (the corresponding interpretation is hence referred to as the “disposable stem-cell model”)	ND	Balordi and Fishell, 2007; Encinas et al., 2011
SGZ	*Nestin:CreERT2* lineage tracing	Up to 1 year	Substantial	Maintenance of NSCs in clones over a year	ND	Balordi and Fishell, 2007; Bonaguidi et al., 2011
SGZ	*Ascl1:CreRT2* + live-imaging (chronic imaging through a window in the overlying cortex)	Up to 2 months	Limited	Progressive consumption of the NSC pool, no return to quiescence upon activation. Compatible with the disposable stem-cell model	ND	Pilz et al., 2018
SGZ	*Hopx:CreERT2*	Up to 12 months	substantial	Quiescent NSCs biased toward neurogenic fates once activated	ND	Berg et al., 2019
SEZ	*Glast:*CreERT2 + confetti reporter mice	4–6 months	Limited	Progressive consumption of the NSC pool with a specific sequence of divisions: few rounds of asymmetric self-renewing divisions symmetric differentiating division	Population asymmetry to maintain neuronal production at the expense of the NSC pool	Calzolari et al., 2015
SEZ	Replication-incompetent avian *RCAS-GFP* retrovirus injected into *hGFAP:Tva* mice	Up to 4 weeks	Limited	Progressive consumption of the NSC pool: 20% of symmetric self-renewing divisions (with return to long-term quiescence), 80% of symmetric differentiating divisions, no asymmetric divisions	Population asymmetry paradigm to balance self-renewal and differentiation of NSCs at the population level	Obernier et al., 2018
SEZ	*Troy: GFPiresCreER* and *Ki67: GFPiresCreER* lineage tracing	Up to 8 months	Substantial self-renewal capacity	NSC fates are chosen stochastically with probabilities inversely correlated with the number of surrounding NSCs	Population asymmetry driven by sensing niche occupancy	Basak et al., 2018

This study raises key questions pertaining to stemness-related fate choices. First, given the relatively uniform generation of neurons across the germinal zone surface and uniform expansion of the NSC population itself, it suggests that NSCs of the different sub-populations are interspersed, neighboring each other across the germinal sheet. This would argue against these different behaviors being controlled exclusively by different extrinsic local cues (such as different local niches), and rather stress the existence of intrinsic control mechanisms encoding one or the other asymmetry behavior. Second, it now pushes to search for molecular signatures of these heterogeneities. In the zebrafish pallium and in the mouse SEZ, it has long been emphasized that NSCs form a very heterogeneous population (Kriegstein and Götz, 2003; März et al., 2010a; Chaker et al., 2016). In the mouse brain and with the recent explosion of scRNA sequencing data, detailed NSC heterogeneities and clusters start to be described (Llorens-Bobadilla et al., 2015; Dulken et al., 2017; Mizrak et al., 2019). The significance and the role of NSC heterogeneities for NSC cell fate choice is not understood, and it will be important to try and overlap this information to transcriptionally identify the distinct NSC pools and directly track their relative contribution to NSC population homeostasis.

## Physiological and Pathological Modulations of Zebrafish Adult Neurogenesis

### Adult Neurogenesis in Zebrafish Responds to and Relays Environmental and Systemic Stimuli

#### Sensory Stimuli, Nutrition, and Stress Exert Parallel Effects on Adult Neurogenesis in Zebrafish and Rodents

**FIGURE 8:**
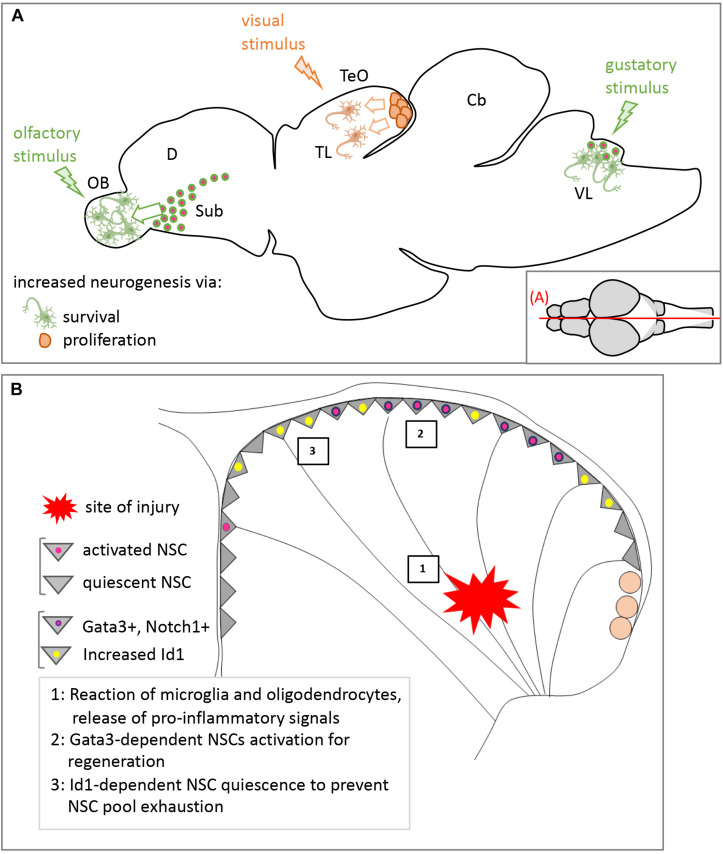
Modulation of adult neurogenesis by external stimuli in zebrafish. **(A)** Influence of sensory stimuli. Representation on a schematic sagittal section of the effect of the different sensory modalities studied to date, which can module either neuronal survival (green) or proliferation of NE progenitors (orange). **(B)** Influence of a mechanical injury on pallial neurogenesis. Representation on a schematic pallial cross-section of the sequence of events following injury (1–3) and the changes in NSC state and gene expression (color-coded). See text for references. Cb, cerebellum; D, dorsal part of the telencephalon; OB, olfactory bulb; Sub, subpallium; TeO, tectum opticum; TL, torus longitudinalis; VL, vagal lobe.

Environmental and systemic factors play an extensive role in modulating neurogenesis. For example, odorant stimuli can be integrated to tune neurogenic output from the SEZ niche in mice. Indeed, there are more newborn neurons in the OB, but not in the hippocampus, of mice reared in an odor-enriched environment (Rochefort et al., 2002), without an increase in proliferation in the SEZ. This suggests that simple sensory stimulation of adult neurogenesis is niche-specific and, in the case of this example, relies on an increase in newborn neuron survival. In teleosts, several neurogenic niches are in regions involved in sensory processing. Among them, the vagal lobe involved in gustation and the olfactory bulb get new neurons from RG NSCs, whereas the caudal periventricular gray zone of the optic tectum and the torus longitudinalis, both involved in visual processing, receive theirs from NE stem cells (Alunni et al., 2010; Sato et al., 2017). Presenting fish with stimuli processed in one of these niches leads to an increase in newborn neurons only in the respective niche and this increase is mediated differently in RG and NE niches: in NE niches, increased neurogenesis relies on an increase in proliferation, while in RG niches it involves an increase in newborn neurons survival (Lindsey et al., 2014), similarly, to OB neurogenesis in mice (Figure 8A).

Nutritional factors also affect rates of neurogenesis in both rodents and zebrafish. Because most experiments involving individual foods were conducted only in mice, we focus here on experimental schemes investigating the effect of global changes of diet on neurogenesis. A high-fat diet and hyperglycemia have generally been linked to decreased hippocampal neurogenesis in rats and mice (Dorsemans et al., 2017a). Similarly, chronic high caloric intake and hyperglycemia lead to diminished proliferation in the forebrain neurogenic niches in zebrafish (Dorsemans et al., 2017b). On the other hand, caloric restriction through intermittent fasting increases the number of BrdU-positive cells after a 4-week chase in the hippocampus. However, equivalent schemes have not been worked out so far in zebrafish, while a global caloric restriction is generally assumed to lead to a decrease in proliferation. An important confounding factor is that the adult zebrafish body and brain keep on growing (Than-Trong et al., 2020), that brain growth notably occurs through the addition of neurons by NSCs, and that this growth is dependent on the quantity of food they receive. This makes it much harder in zebrafish than in rodents to ascertain whether changes in proliferation rates of NSCs are due to a specific regulation of NSC behavior or dictated by organism growth when these changes go in the same direction. Therefore, while the zebrafish can be used to investigate metabolic control of neurogenesis due to conservation of physiological responses to metabolic imbalances (Craig and Moon, 2011), there is still a need to work out experimental conditions before using it as a model for interventions that can interfere with its growth.

Finally, factors influencing emotional states also have consequences on neurogenesis. In particular, chronic high stress induced by social isolation decreases proliferation in the hippocampus of mice (Ibi et al., 2008) and non-human primates (Cinini et al., 2014) as well as in the forebrain of zebrafish (Tea et al., 2019).

These results together illustrate that adult neurogenesis in zebrafish is sensitive to environmental cues. Whether and how newly generated neurons relay some important measure of these stimuli, or convey some physiological response, remains to be shown.

#### Hormonal Regulation of Zebrafish Adult Neurogenesis

The environmental cues discussed above can be relayed to NSCs or adult newborn neurons via the activity of neurons contacting germinal zones. In many cases, however, they are also mediated by hormones. Among them, steroid hormones have been the subject of much focus. Glucocorticoids are elevated in response to stress as well as under high-fat, high-sugar and hyper caloric diets, and appear to be the cause of the reduced neurogenesis in these cases. Of note, in many species including amphibians, rodents and birds, the main glucocorticoid is corticosterone whereas in humans and teleost fish the main glucocorticoid is cortisol (which differs from corticosterone by the presence of one additional hydroxyl group), making zebrafish a particularly attractive model to study the effects of glucocorticoids on adult neurogenesis.

Sex steroids and in particular estrogens have also been extensively studied for their role in modulating adult neurogenesis. In the end, the nature of their involvement seems to not be conserved between species, even among rodents (Tanapat et al., 1999, 2005; Ormerod and Galea, 2001; Ormerod et al., 2003; Brock et al., 2010). In zebrafish, experimentally increasing estradiol levels decreases proliferation in the subpallium and some pallial subdivisions (Dl but not Dm) (Diotel et al., 2013; Makantasi and Dermon, 2014). An important peculiarity of teleosts when it comes to estrogen signaling is the duplication of the *cyp19a1* gene coding for aromatase. In mammals, aromatase is expressed in the gonads and in a few neurons, whereas in zebrafish, aromatase A is expressed in the gonads while aromatase B is expressed at high levels in radial glia (Pellegrini et al., 2007). There, it could contribute to local estrogen synthesis, and this has been proposed to actively suppress proliferation in some neurogenic niches, in particular at the junction between olfactory bulbs and telencephalon and in the pallial region (Diotel et al., 2013).

Several other hormones are known to regulate neurogenesis in rodents such as Ghrelin, Thyroid hormones, Adiponectin and Androgens, however, their action has not yet been investigated in zebrafish. Mapping the expression of their receptors in the brain could constitute a first step in determining whether these regulations are conserved across species (Rastegar et al., 2019).

### Zebrafish Adult Pallial NSCs Contribute Actively to Neuronal Regeneration

#### Pallial NSCs Are Activated for Regeneration Upon Mechanical Lesion

Perhaps the most well-known feature of zebrafish adult neurogenesis is its ability to contribute to neuronal regeneration after a brain injury contrary to mammals. In rodents and non-human primates, traumatic or excitotoxic brain injuries can increases NSC proliferation. However, neuron generation is inefficient: a fraction of NSC divisions is gliogenic and generates astrocytes, neuroblasts often fail to migrate toward the site of injury, and the neurogenesis process is usually not followed by functional integration of the new neurons (Skaggs et al., 2014) -although with a few exceptions (Nakatomi et al., 2002; Sirko et al., 2013; Magnusson et al., 2014)-. Upon injury in mammals, inflammation triggers the activation of reactive astrocytes and the formation of a glial scar which prevents regeneration (März et al., 2011). On the opposite, in the mechanically injured zebrafish pallium, nearby RG cells in the ventricular zone quickly increase their proliferation rate, reaching a peak at 7 days post-injury (dpi) before progressively returning to baseline. Genetic or BrdU-mediated tracing indicated that this allows for the production of newborn neurons that migrate to the site of injury and get synaptically connected. These neurons can survive for at least 3 months, leading to wound closure without formation of a glial scar (Kroehne et al., 2011; März et al., 2011; Baumgart et al., 2012). Their identity, however, remains to be described – notably to determine whether it matches that of the lesioned neurons-. Functional recovery also needs to be assessed, although this may prove a difficult task given that functional pallial neuroanatomy is not precise at this point. Overall, a huge efforts needs to be made to map functional circuits and their markers in adult zebrafish.

Similarly, the initial response to pallial injury in zebrafish is an activation of microglia and oligodendrocytes surrounding the site of injury, together with pro-inflammatory signals, but contrary to the situation in rodents, they act to promote regeneration. Expression of *cysteinyl leukotriene receptor 1* (*cysltr1*) is upregulated especially close to the site of injury and, upon binding its ligand CysLT1, triggers the expression of Gata3 (Kyritsis et al., 2012), a transcription factor which is normally not (or lowly) expressed under physiological condition (Kizil et al., 2012c). Expression of *gata3* is necessary for injury-induced NSC proliferation in the pallium, and experimental stimulation of CysLTr1 is in turn sufficient and necessary to induce *gata3* and increase NSC proliferation (Figure 8B). Other proinflammatory signals probably also play a role as expression of *cxcr5* is also increased after injury and its blockade reduces the regenerative response (Kizil et al., 2012a). Moreover, while inflammation plays an essential role in initiating the regenerative response, other signaling pathways are also necessary for it to reach its full extent. Indeed, blocking FGF signaling after injury reduces the upregulation of *gata3* and proliferation of nearby NSCs (Kizil et al., 2012c). Regulatory mechanisms involved in controlling neurogenesis in physiological conditions are also essential in response to injury. After injury, BDNF is upregulated in the surrounding newborn and mature neurons for up to 15 days and acts through its receptor TrkB to promote NSC proliferation. Likewise, the expression of proteins involved in the Notch signaling pathway is modified upon lesion, and non-selectively inhibiting it with the gamma-secretase inhibitor DAPT decreases the magnitude of the response in NSCs. Moreover, division modes upon injury favor a more neurogenic fate at the expense of self-renewal, which risks leading to depletion of the NSC pool (Barbosa et al., 2015a). The upregulation of Notch1 or Notch3, which promote stemness and quiescence (Alunni et al., 2013; Than-Trong et al., 2018) could be a way to counteract this depletion. This was formally demonstrated to be a function of Id1, which is also upregulated upon injury independently of inflammatory signals and of Notch signaling and mitigates the proliferation of NSCs upon injury (Rodriguez-Viales et al., 2015).

The improved regeneration in zebrafish thus appears to be in part due to the absence of parenchymal astrocytes that generate a glial scar and a different management of the inflammation response that recedes faster in zebrafish (Kizil et al., 2012b). In addition, the “protection” of a subset of NSCs from the regenerative response might be relevant to also maintain physiological neurogenesis post-lesion. Getting a full picture of those differences represents a promising avenue to better understand how to promote neuronal regeneration for therapeutic purposes.

#### Pallial NSCs Are Activated for Regeneration Upon Neuronal Alzheimer-Like Degeneration

One of the regions affected early in Alzheimer’s disease (AD) is the hippocampus, and this hippocampal degeneration is thought to underlie the memory loss symptoms as well as the visuo-spatial disorientation that appear from the early stages of the disease. Stimulating hippocampal neurogenesis in order to regenerate the lost neurons and rescue hippocampal function is thus considered a potential therapy to alleviate the disease. In mouse models of AD where the disease is replicated by inducing the formation of amyloid beta plaques in the brain, and in samples from AD patients, the production of newborn neurons appears increased (Jin et al., 2004; Gan et al., 2008; Unger et al., 2016). However, the NSCs themselves also seem affected by the disease, leading to depletion of the NSC pool (Moreno-Jiménez et al., 2019). Recently the Kizil group proposed a zebrafish AD model using amyloid-b42 injections into the adult pallium (Bhattarai et al., 2016). While the relevance of such a model to the human disease still needs to be fully validated, the results suggest that NSCs proliferate in response to amyloidopathies through IL4 signaling but that not all NSCs respond similarly (Bhattarai et al., 2016; Cosacak et al., 2019). Understanding the bases for these differences will be an important point for future studies.

## Conclusion

The location and efficiency of adult neurogenesis domains, under physiological or pathological conditions, vary greatly between vertebrate species. The mechanistic reasons for these differences largely remain to be understood, and comparative approaches are powerful ways toward this goal. As illustrated in this review, the zebrafish adult pallium offers novel perspectives to dive into the fundamental properties of adult telencephalic NSCs. These are linked in particular with unprecedented possibilities to record the behavior of NSCs in their niche (such as intravital imaging methods), and with the existence of unique physiological contexts (such as regeneration), associated with a vast repertoire of NSC properties that can be mechanistically matched or contrasted with rodent NSCs. Princeps discoveries on adult NSC quiescence and population dynamics were obtained in zebrafish with strong applicability potential to rodents, and continuation of such synergistic work will undoubtedly help progress in the field.

## Author Contributions

ML, LM, DM, and LB-C wrote the review. LB-C coordinated the process. All authors contributed to the article and approved the submitted version.

## Conflict of Interest

The authors declare that the research was conducted in the absence of any commercial or financial relationships that could be construed as a potential conflict of interest.

## References

[B1] Adolf B. Chapouton P. Lam C. S. Topp S. Tannhäuser B. Strähle U. (2006). Conserved and acquired features of adult neurogenesis in the zebrafish telencephalon. *Dev. Biol.* 295 278–293. 10.1016/j.ydbio.2006.03.023 16828638

[B2] Aguirre A. Rubio M. E. Gallo V. (2010). Notch and EGFR pathway interaction regulates neural stem cell number and self-renewal. *Nature* 467 323–327. 10.1038/nature09347 20844536 PMC2941915

[B3] Altman J. Das G. D. (1965). Autoradiographic and histological evidence of postnatal hippocampal neurogenesis in rats. *J. Comp. Neurol.* 124 319–335. 10.1002/cne.901240303 5861717

[B4] Alunni A. Hermel J. M. Heuzé A. Bourrat F. Jamen F. Joly J. S. (2010). Evidence for neural stem cells in the Medaka optic tectum proliferation zones. *Dev. Neurobiol.* 70 693–713. 10.1002/dneu.20799 20506557

[B5] Alunni A. Krecsmarik M. Bosco A. Galant S. Pan L. Moens C. B. (2013). Notch3 signaling gates cell cycle entry and limits neural stem cell amplification in the adult pallium. *Development* 140 3335–3347. 10.1242/dev.095018 23863484 PMC3737716

[B6] Anacker C. Hen R. (2017). Adult hippocampal neurogenesis and cognitive flexibility-linking memory and mood. *Nat. Rev. Neurosci.* 18 335–346. 10.1038/nrn.2017.45 28469276 PMC6261347

[B7] Anand S. K. Mondal A. C. (2017). Cellular and molecular attributes of neural stem cell niches in adult zebrafish brain. *Dev. Neurobiol.* 77 1188–1205. 10.1002/dneu.22508 28589616

[B8] Andersen J. Urbán N. Achimastou A. Ito A. Simic M. Ullom K. (2014). A transcriptional mechanism integrating inputs from extracellular signals to activate hippocampal stem cells. *Neuron* 83 1085–1097. 10.1016/j.neuron.2014.08.004 25189209 PMC4157576

[B9] Andreu-Agulló C. Morante-Redolat J. M. Delgado A. C. Farias I. (2009). Vascular niche factor PEDF modulates notch-dependent stemness in the adult subependymal zone. *Nat. Neurosci.* 12 1514–1523. 10.1038/nn.2437 19898467

[B10] Bai G. Sheng N. Xie Z. Bian W. Yokota Y. Benezra R. (2007). Id sustains hes1 expression to inhibit precocious neurogenesis by releasing negative Autoregulation of Hes1. *Dev. Cell* 13 283–297. 10.1016/j.devcel.2007.05.014 17681138

[B11] Balordi F. Fishell G. (2007). Mosaic removal of hedgehog signaling in the adult SVZ reveals that the residual wild-type stem cells have a limited capacity for self-renewal. *J. Neurosci.* 27 14248–14259. 10.1523/jneurosci.4531-07.2007 18160632 PMC6673442

[B12] Barbosa J. S. Sanchez-gonzalez R. Di Giaimo R. Baumgart E. V. Theis F. J. Ninkovic J. (2015a). Live imaging of adult neural stem cell behavior in the intact and injured zebrafish brain. *Science* 65 61–65. 10.1126/science.aaa2729 25977550

[B13] Barbosa J. S. Sanchez-Gonzalez R. Di Giaimo R. Baumgart E. V. Theis F. J. Götz M. (2015b). Neurodevelopment. Live imaging of adult neural stem cell behavior in the intact and injured zebrafish brain. *Science* 348 789–793. 10.1126/science.aaa2729 25977550

[B14] Basak O. Giachino C. Fiorini E. MacDonald H. R. Taylor V. (2012). Neurogenic Subventricular zone stem/progenitor cells are notch1-dependent in their active but not Quiescent State. *J. Neurosci.* 32 5654–5666. 10.1523/jneurosci.0455-12.2012 22514327 PMC6703480

[B15] Basak O. Krieger T. G. Muraro M. J. Wiebrands K. Stange D. E. Frias-Aldeguer J. (2018). Troy+ brain stem cells cycle through quiescence and regulate their number by sensing niche occupancy. *Proc. Natl. Acad. Sci. U.S.A.* 115 E610–E619. 29311336 10.1073/pnas.1715911114 PMC5789932

[B16] Baser A. Skabkin M. Kleber S. Dang Y. Gülcüler Balta G. S. Kalamakis G. (2019). Onset of differentiation is post-transcriptionally controlled in adult neural stem cells. *Nature* 566 100–104. 10.1038/s41586-019-0888-x 30700908

[B17] Bast L. Calzolari F. Strasser M. K. Hasenauer J. Theis F. J. Ninkovic J. (2018). Increasing neural stem cell division asymmetry and quiescence are predicted to contribute to the age-related decline in neurogenesis. *Cell Rep.* 25 3231–3240. 30566852 10.1016/j.celrep.2018.11.088

[B18] Baumgart E. V. Barbosa J. S. Bally-cuif L. Götz M. Ninkovic J. (2012). Stab wound injury of the zebrafish telencephalon: a model for comparative analysis of reactive gliosis. *Glia* 60 343–357. 10.1002/glia.22269 22105794

[B19] Bayer S. A. (1985). Neuron production in the hippocampus and olfactory bulb of the adult rat brain: addition or replacement? *Ann. N. Y. Acad. Sci.* 457 163–172. 10.1111/j.1749-6632.1985.tb20804.x 3868311

[B20] Berberoglu M. A. Dong Z. Li G. Zheng J. Trejo Martinez L. D. C. G. Peng J. (2014). Heterogeneously expressed fezf2 patterns gradient notch activity in balancing the quiescence, proliferation, and differentiation of adult neural stem cells. *J. Neurosci.* 34 13911–13923. 10.1523/jneurosci.1976-14.2014 25319688 PMC4198537

[B21] Berg D. A. Kirkham M. Beljajeva A. Knapp D. Habermann B. Ryge J. (2010). Efficient regeneration by activation of neurogenesis in homeostatically quiescent regions of the adult vertebrate brain. *Development* 137 4127–4134. 10.1242/dev.055541 21068061

[B22] Berg D. A. Su Y. Jimenez-Cyrus D. Patel A. Huang N. Morizet D. (2019). A common embryonic origin of stem cells drives developmental and adult neurogenesis. *Cell* 177 654–668. 30929900 10.1016/j.cell.2019.02.010 PMC6496946

[B23] Bhattarai P. Thomas A. K. Cosacak M. I. Papadimitriou C. Mashkaryan V. Froc C. (2016). IL4/STAT6 signaling activates neural stem cell proliferation and neurogenesis upon amyloid-β42 aggregation in Adult Zebrafish brain. *Cell Rep.* 17 941–948. 10.1016/j.celrep.2016.09.075 27760324

[B24] Blanpain C. Simons B. D. (2013). Unravelling stem cell dynamics by lineage tracing. *Nat. Rev. Mol. Cell Biol.* 14 489–502. 10.1038/nrm3625 23860235

[B25] Blažek R. Polačik M. Reichard M. (2013). Rapid growth, early maturation and short generation time in African annual fishes. *EvoDevo* 4:24. 10.1186/2041-9139-4-24 24007640 PMC3844391

[B26] Blomfield I. M. Rocamonde B. del Mar Masdeu M. Mulugeta E. Vaga S. van den Berg D. L. C. (2019). Id4 promotes the elimination of the pro-activation factor ascl1 to maintain quiescence of adult hippocampal stem cells. *eLife* 8:e48561. 10.7554/eLife.48561 PMC6805120 31552825

[B27] Boareto M. Iber D. Taylor V. (2017). Differential interactions between Notch and ID factors control neurogenesis by modulating Hes factor autoregulation. *Development* 144 3465–3474. 10.1242/dev.152520 28974640 PMC5665482

[B28] Bonaguidi M. A. Peng C.-Y. McGuire T. Falciglia G. Gobeske K. T. Czeisler C. (2008). Noggin expands neural stem cells in the adult hippocampus. *J. Neurosci.* 28 9194–9204. 10.1523/jneurosci.3314-07.2008 18784300 PMC3651371

[B29] Bonaguidi M. A. Song J. Ming G. L. Song H. (2012). A unifying hypothesis on mammalian neural stem cell properties in the adult hippocampus. *Curr. Opin. Neurobiol.* 22 754–761. 10.1016/j.conb.2012.03.013 22503352 PMC3415562

[B30] Bonaguidi M. A. Wheeler M. A. Shapiro J. S. Stadel R. P. Sun G. J. Ming G. L. (2011). In vivo clonal analysis reveals self-renewing and multipotent adult neural stem cell characteristics. *Cell* 145 1142–1155. 10.1016/j.cell.2011.05.024 21664664 PMC3124562

[B31] Brock O. Keller M. Veyrac A. Douhard Q. Bakker J. (2010). Short term treatment with estradiol decreases the rate of newly generated cells in the subventricular zone and main olfactory bulb of adult female mice. *Neuroscience* 166 368–376. 10.1016/j.neuroscience.2009.12.050 20045446

[B32] Byrd C. A. Brunjes P. C. (2001). Neurogenesis in the olfactory bulb of adult zebrafish. *Neuroscience* 105 793–801. 10.1016/s0306-4522(01)00215-9 11530218

[B33] Calegari F. Huttner W. B. (2003). An inhibition of cyclin-dependent kinases that lengthens, but does not arrest, neuroepithelial cell cycle induces premature neurogenesis. *J. Cell Sci.* 116 4947–4955. 10.1242/jcs.00825 14625388

[B34] Calzolari F. Michel J. Baumgart E. V. Theis F. Götz M. Ninkovic J. (2015). Fast clonal expansion and limited neural stem cell self-renewal in the adult subependymal zone. *Nat. Neurosci.* 18 490–492. 10.1038/nn.3963 25730673

[B35] Castro D. S. Martynoga B. Parras C. Ramesh V. Pacary E. Johnston C. (2011). A novel function of the proneural factor Ascl1 in progenitor proliferation identified by genome-wide characterization of its targets. *Genes Dev.* 25 930–945. 10.1101/gad.627811 21536733 PMC3084027

[B36] Chaker Z. Codega P. Doetsch F. (2016). A mosaic world: puzzles revealed by adult neural stem cell heterogeneity. *Wiley Interdiscip. Rev. Dev. Biol.* 5 640–658. 10.1002/wdev.248 27647730 PMC5113677

[B37] Chang C. D. Ottavio L. Travali S. Lipson K. E. Baserga R. (1990). Transcriptional and posttranscriptional regulation of the proliferating cell nuclear antigen gene. *Mol. Cell. Biol.* 10 3289–3296. 10.1128/mcb.10.7.3289 1972540 PMC360744

[B38] Chapouton P. Skupien P. Hesl B. Coolen M. Moore J. C. Madelaine R. (2010). Notch activity levels control the balance between quiescence and recruitment of adult neural stem cells. *J. Neurosci.* 30 7961–7974. 10.1523/jneurosci.6170-09.2010 20534844 PMC6632678

[B39] Chen X. Hartman A. Guo S. (2015). Choosing cell fate through a dynamic cell cycle. *Curr. Stem Cell Rep.* 1 129–138. 10.1007/s40778-015-0018-0 28725536 PMC5487535

[B40] Cinini S. M. Barnabe G. F. Galvão-Coelho N. de Medeiros M. A. Perez-Mendes P. Sousa M. B. C. (2014). Social isolation disrupts hippocampal neurogenesis in young non-human primates. *Front. Neurosci.* 8:45. 10.3389/fnins.2014.00045 24733997 PMC3973924

[B41] Codega P. Silva-Vargas V. Paul A. Maldonado-Soto A. R. R. DeLeo A. M. M. Pastrana E. (2014). Prospective identification and purification of quiescent adult neural stem cells from their *in vivo* niche. *Neuron* 82 545–559. 10.1016/j.neuron.2014.02.039 24811379 PMC4360885

[B42] Coolen M. Labusch M. Mannioui A. Bally-Cuif L. (2020). Mosaic heterochrony in neural progenitors sustains accelerated brain growth and neurogenesis in the Juvenile Killifish *N. furzeri*. *Curr. Biol.* 30 736–745. 32004451 10.1016/j.cub.2019.12.046 PMC7040570

[B43] Cosacak M. I. Bhattarai P. Reinhardt S. Petzold A. Dahl A. Zhang Y. (2019). Single-cell transcriptomics analyses of neural stem cell heterogeneity and contextual plasticity in a Zebrafish brain model of amyloid toxicity. *Cell Rep.* 27 1307–1318. 31018142 10.1016/j.celrep.2019.03.090

[B44] Craig P. M. Moon T. W. (2011). Fasted zebrafish mimic genetic and physiological responses in mammals: a model for obesity and diabetes? *Zebrafish* 8 109–117. 10.1089/zeb.2011.0702 21854210

[B45] Dambroise E. Simion M. Bourquard T. Bouffard S. Rizzi B. Jaszczyszyn Y. (2017). Postembryonic fish brain proliferation zones exhibit neuroepithelial-type gene expression profile. *Stem Cells* 35 1505–1518. 10.1002/stem.2588 28181357

[B46] de Oliveira-Carlos V. Ganz J. Hans S. Kaslin J. Brand M. (2013). Notch receptor expression in neurogenic regions of the adult Zebrafish brain. *PLoS One* 8:e73384. 10.1371/journal.pone.0073384 24039926 PMC3767821

[B47] Diotel N. Beil T. Strähle U. Rastegar S. (2015). Differential expression of id genes and their potential regulator znf238 in zebrafish adult neural progenitor cells and neurons suggests distinct functions in adult neurogenesis. *Gene Expr. Patterns* 19 1–13. 10.1016/j.gep.2015.05.004 26107416

[B48] Diotel N. Vaillant C. Gabbero C. Mironov S. Fostier A. Gueguen M. M. (2013). Effects of estradiol in adult neurogenesis and brain repair in zebrafish. *Horm. Behav.* 63 193–207. 10.1016/j.yhbeh.2012.04.003 22521210

[B49] Dirian L. Galant S. Coolen M. Chen W. Bedu S. Houart C. (2014). Spatial regionalization and heterochrony in the formation of adult pallial neural stem cells. *Dev. Cell* 30 123–136. 10.1016/j.devcel.2014.05.012 25017692

[B50] Doe C. Q. (2017). Temporal patterning in the Drosophila CNS. *Annu. Rev. Cell Dev. Biol.* 33 219–240. 10.1146/annurev-cellbio-111315-125210 28992439

[B51] Dong Z. Yang N. Yeo S.-Y. Chitnis A. Guo S. (2012). Intralineage directional Notch signaling regulates self-renewal and differentiation of asymmetrically dividing radial glia. *Neuron* 74 65–78. 10.1016/j.neuron.2012.01.031 22500631 PMC3466114

[B52] Dorsemans A. C. Couret D. Hoarau A. Meilhac O. Lefebvre d’Hellencourt C. Diotel N. (2017a). Diabetes, adult neurogenesis and brain remodeling: new insights from rodent and zebrafish models. *Neurogenesis* 4:e1281862. 10.1080/23262133.2017.1281862 28439518 PMC5397121

[B53] Dorsemans A. C. Soulé S. Weger M. Bourdon E. Lefebvre d’Hellencourt C. Meilhac O. (2017b). Impaired constitutive and regenerative neurogenesis in adult hyperglycemic zebrafish. *J. Comp. Neurol.* 525 442–458. 10.1002/cne.24065 27339277

[B54] Dranovsky A. Picchini A. M. Moadel T. Sisti A. C. Yamada A. Kimura S. (2011). Experience dictates stem cell fate in the adult hippocampus. *Neuron* 70 908–923. 10.1016/j.neuron.2011.05.022 21658584 PMC3124009

[B55] Dray N. Bedu S. Vuillemin N. Alunni A. Coolen M. Krecsmarik M. (2015). Large-scale live imaging of adult neural stem cells in their endogenous niche. *Development* 142 3592–3600. 10.1242/dev.123018 26395477 PMC4631764

[B56] Dulken B. W. Leeman D. S. Boutet S. C. Hebestreit K. Brunet A. Blurb E. (2017). Single cell transcriptomic analysis defines heterogeneity and transcriptional dynamics in the adult neural stem cell lineage. *Cell Rep.* 1712 777–790. 10.1016/j.celrep.2016.12.060 28099854 PMC5269583

[B57] Ehm O. Göritz C. Covic M. Schäffner I. Schwarz T. J. Karaca E. (2010). RBPJkappa-dependent signaling is essential for long-term maintenance of neural stem cells in the adult hippocampus. *J. Neurosci.* 30 13794–13807. 10.1523/jneurosci.1567-10.2010 20943920 PMC6633732

[B58] Encinas J. M. Michurina T. V. Peunova N. Park J. H. Tordo J. Peterson D. A. (2011). Division-coupled astrocytic differentiation and age-related depletion of neural stem cells in the adult hippocampus. *Cell Stem Cell* 8 566–579. 10.1016/j.stem.2011.03.010 21549330 PMC3286186

[B59] Engler A. Rolando C. Giachino C. Saotome I. Erni A. Brien C. (2018). Notch2 signaling maintains NSC quiescence in the murine ventricular-Subventricular zone. *Cell Rep.* 22 992–1002. 10.1016/j.celrep.2017.12.094 29386140

[B60] Eriksson P. S. Perfilieva E. Björk-Eriksson T. Alborn A. M. Nordborg C. Peterson D. A. (1998). Neurogenesis in the adult human hippocampus. *Nat. Med.* 4 1313–1317. 9809557 10.1038/3305

[B61] Fan X. Xiong Y. Wang Y. (2019). A reignited debate over the cell(s) of origin for glioblastoma and its clinical implications. *Front. Med.* 13:531–539. 10.1007/s11684-019-0700-1 31313083

[B62] Ferron S. R. Pozo N. Laguna A. Aranda S. Porlan E. Moreno M. (2010). Regulated segregation of kinase Dyrk1A during asymmetric neural stem cell division is critical for EGFR-mediated biased signaling. *Cell Stem Cell* 7 367–379. 10.1016/j.stem.2010.06.021 20804972

[B63] Folgueira M. Bayley P. Navratilova P. Becker T. S. Wilson S. W. Clarke J. D. W. (2012). Morphogenesis underlying the development of the everted teleost telencephalon. *Neural Dev.* 7:32. 10.1186/1749-8104-7-32 22989074 PMC3520737

[B64] Fuentealba L. C. Obernier K. Alvarez-Buylla A. (2012). Adult neural stem cells bridge their niche. *Cell Stem Cell* 10 698–708. 10.1016/j.stem.2012.05.012 22704510 PMC3726005

[B65] Furlan G. Cuccioli V. Vuillemin N. Dirian L. Muntasell A. J. Coolen M. (2017). Life-long neurogenic activity of individual neural stem cells and continuous growth establish an outside-in architecture in the Teleost Pallium. *Curr. Biol.* 27 3288–3301.e3. 10.1016/j.cub.2017.09.052 29107546 PMC5678050

[B66] Furutachi S. Miya H. Watanabe T. Kawai H. Yamasaki N. Harada Y. (2015). Slowly dividing neural progenitors are an embryonic origin of adult neural stem cells. *Nat. Neurosci.* 18 657–665. 10.1038/nn.3989 25821910

[B67] Gan L. Qiao S. Lan X. Chi L. Luo C. Lien L. (2008). Neurogenic responses to amyloid-beta plaques in the brain of Alzheimer’s disease-like transgenic (pPDGF-APPSw,Ind) mice. *Neurobiol. Dis.* 29 71–80. 10.1016/j.nbd.2007.08.002 17916429 PMC2180424

[B68] Ganz J. Kaslin J. Hochmann S. Freudenreich D. Brand M. (2010). Heterogeneity and Fgf dependence of adult neural progenitors in the zebrafish telencephalon. *Glia* 58 1345–1363. 10.1002/glia.21012 20607866

[B69] Ganz J. Kroehne V. Freudenreich D. Machate A. Geffarth M. Braasch I. (2015). Subdivisions of the adult zebrafish pallium based on molecular marker analysis. *F1000Res.* 520 633–655. 10.1002/cne.22757 25713698 PMC4335597

[B70] Giachino C. Taylor V. (2014). Notching up neural stem cell homogeneity in homeostasis and disease. *Front. Neurosci.* 8:32. 10.3389/fnins.2014.00032 24611040 PMC3933793

[B71] Goldman S. A. Nottebohm F. (1983). Neuronal production, migration, and differentiation in a vocal control nucleus of the adult female canary brain (learning/neurogenesis/neuronal death/glial cells/endothelial cells). *Proc. Nati. Acad. Sci. U.S.A.* 80 2390–2394. 10.1073/pnas.80.8.2390 6572982 PMC393826

[B72] Götz M. Nakafuku M. Petrik D. (2016). Neurogenesis in the developing and adult brain-similarities and key differences. *Cold Spring Harb. Perspect. Biol.* 8:a018853. 10.1101/cshperspect.a018853 27235475 PMC4930921

[B73] Grandel H. Kaslin J. Ganz J. Wenzel I. Brand M. (2006). Neural stem cells and neurogenesis in the adult zebrafish brain: origin, proliferation dynamics, migration and cell fate. *Dev. Biol.* 295 263–277. 10.1016/j.ydbio.2006.03.040 16682018

[B74] Guesmi K. Abdeladim L. Tozer S. Mahou P. Kumamoto T. Jurkus K. (2018). Dual-color deep-tissue three-photon microscopy with a multiband infrared laser. *Light Sci. Appl.* 7 12 10.1017/s1551929512000430 PMC6107000 30839589

[B75] Harris L. Zalucki O. Clément O. Fraser J. Matuzelski E. Oishi S. (2018). Neurogenic differentiation by hippocampal neural stem and progenitor cells is biased by NFIX expression. *Development* 145:dev155689. 10.1242/dev.155689 29437824

[B76] Hevner R. F. (2019). Intermediate progenitors and Tbr2 in cortical development. *J. Anat.* 235 616–625. 10.1111/joa.12939 30677129 PMC6656625

[B77] Hochgerner H. Zeisel A. Lönnerberg P. Linnarsson S. (2018). Conserved properties of dentate gyrus neurogenesis across postnatal development revealed by single-cell RNA sequencing. *Nat. Neurosci.* 21 290–299. 10.1038/s41593-017-0056-2 29335606

[B78] Huttner W. B. Kosodo Y. (2005). Symmetric versus asymmetric cell division during neurogenesis in the developing vertebrate central nervous system. *Curr. Opin. Cell Biol.* 17 648–657. 10.1016/j.ceb.2005.10.005 16243506

[B79] Ibi D. Takuma K. Koike H. Mizoguchi H. Tsuritani K. Kuwahara Y. (2008). Social isolation rearing-induced impairment of the hippocampal neurogenesis is associated with deficits in spatial memory and emotion-related behaviors in juvenile mice. *J. Neurochem.* 105 921–932. 10.1111/j.1471-4159.2007.05207.x 18182044

[B80] Imayoshi I. Sakamoto M. Yamaguchi M. Mori K. Kageyama R. (2010). Essential roles of notch signaling in maintenance of neural stem cells in developing and adult brains. *J. Neurosci.* 30 3489–3498. 10.1523/jneurosci.4987-09.2010 20203209 PMC6634119

[B81] Jin K. Peel A. L. Mao X. O. Xie L. Cottrell B. A. Henshall D. C. (2004). Increased hippocampal neurogenesis in Alzheimer’s disease. *Proc. Natl. Acad. Sci. U.S.A.* 101 343–347. 14660786 10.1073/pnas.2634794100 PMC314187

[B82] Jorgensen C. (2018). Adult mammalian neurogenesis and motivated behaviors. *Integr. Zool.* 16 655–672. 10.1111/1749-4877.12335 29851231

[B83] Kalamakis G. Brüne D. Ravichandran S. Bolz J. Fan W. Ziebell F. (2019). Quiescence modulates stem cell maintenance and regenerative capacity in the aging brain. *Cell* 176 1407–1419.e14. 10.1016/j.cell.2019.01.040 30827680

[B84] Katz S. Cussigh D. Urbán N. Blomfield I. Guillemot F. Bally-Cuif L. (2016). A nuclear role for miR-9 and argonaute proteins in balancing quiescent and activated neural stem cell states. *Cell Rep.* 17 1383–1398. 10.1016/j.celrep.2016.09.088 27783951 PMC5098119

[B85] Kawaguchi D. Furutachi S. Kawai H. Hozumi K. Gotoh Y. (2013). Dll1 maintains quiescence of adult neural stem cells and segregates asymmetrically during mitosis. *Nat. Commun.* 4:1880. 10.1038/ncomms2895 PMC3675328 23695674

[B86] Kawai H. Kawaguchi D. Kuebrich B. D. Kitamoto T. Yamaguchi M. Gotoh Y. (2017). Area-specific regulation of quiescent neural stem cells by Notch3 in the adult mouse subependymal zone. *J. Neurosci.* 37 11867–11880. 10.1523/jneurosci.0001-17.2017 29101245 PMC6596834

[B87] Kishimoto N. Alfaro-Cervello C. Shimizu K. Asakawa K. Urasaki A. Nonaka S. (2011). Migration of neuronal precursors from the telencephalic ventricular zone into the olfactory bulb in adult zebrafish. *J. Comp. Neurol.* 519 3549–3565. 10.1002/cne.22722 21800305

[B88] Kizil C. Dudczig S. Kyritsis N. Machate A. Blaesche J. Kroehne V. (2012a). The chemokine receptor cxcr5 regulates the regenerative neurogenesis response in the adult zebrafish brain. *Neural Dev.* 7:27. 10.1186/1749-8104-7-27 22824261 PMC3441421

[B89] Kizil C. Kaslin J. Kroehne V. Brand M. (2012b). Adult neurogenesis and brain regeneration in zebrafish. *Dev. Neurobiol.* 72 429–461. 10.1002/dneu.20918 21595047

[B90] Kizil C. Kyritsis N. Dudczig S. Kroehne V. Freudenreich D. Kaslin J. (2012c). Regenerative neurogenesis from neural progenitor cells requires injury-induced expression of Gata3. *Dev. Cell* 23 1230–1237. 10.1016/j.devcel.2012.10.014 23168169

[B91] Knoblich J. A. (2008). Mechanisms of asymmetric stem cell division. *Cell* 132 583–597. 10.1016/j.cell.2008.02.007 18295577

[B92] Kressmann S. Campos C. Castanon I. Fürthauer M. González-Gaitán M. (2015). Directional Notch trafficking in Sara endosomes during asymmetric cell division in the spinal cord. *Nat. Cell Biol.* 17 333–339. 10.1038/ncb3119 25706234

[B93] Kriegstein A. R. Götz M. (2003). Radial glia diversity: a matter of cell fate. *Glia* 43 37–43. 10.1002/glia.10250 12761864

[B94] Kroehne V. Freudenreich D. Hans S. Kaslin J. Brand M. (2011). Regeneration of the adult zebrafish brain from neurogenic radial glia-type progenitors. *Development* 138 4831–4841. 10.1242/dev.072587 22007133

[B95] Kyritsis N. Kizil C. Zocher S. Kroehne V. Kaslin J. Freudenreich D. (2012). Acute inflammation initiates the regenerative response in the adult zebrafish brain. *Science* 338 1353–1356. 10.1126/science.1228773 23138980

[B96] Lal P. Tanabe H. Suster M. L. Ailani D. Kotani Y. Muto A. (2018). Identification of a neuronal population in the telencephalon essential for fear conditioning in zebrafish. *BMC Biol.* 16:45. 10.1186/s12915-018-0502-y 29690872 PMC5978991

[B97] Lange C. Rost F. Machate A. Reinhardt S. Lesche M. Weber A. (2020). Single cell sequencing of radial glia progeny reveals the diversity of newborn neurons in the adult zebrafish brain. *Development* 147:dev185595. 10.1242/dev.185595 31908317 PMC6983714

[B98] Lavado A. Oliver G. (2014). Jagged1 is necessary for postnatal and adult neurogenesis in the dentate gyrus. *Dev. Biol.* 388 11–21. 10.1016/j.ydbio.2014.02.004 24530424 PMC4009513

[B99] Levison S. W. Goldman J. E. (1993). Both oligodendrocytes and astrocytes develop from progenitors in the subventricular zone of postnatal rat forebrain. *Neuron* 10 201–212. 10.1016/0896-6273(93)90311-e 8439409

[B100] Li G. Fang L. Fernández G. Pleasure S. J. (2013). The ventral hippocampus is the embryonic origin for adult neural stem cells in the Dentate Gyrus. *Neuron* 78 658–672. 10.1016/j.neuron.2013.03.019 23643936 PMC3669230

[B101] Lim D. A. Tramontin A. D. Trevejo J. M. Herrera D. G. García-Verdugo J. M. Alvarez-Buylla A. (2000). Noggin antagonizes BMP signaling to create a niche for adult neurogenesis. *Neuron* 28 713–726. 10.1016/s0896-6273(00)00148-3 11163261

[B102] Lindsey B. W. Di Donato S. Kaslin J. Tropepe V. (2014). Sensory-specific modulation of adult neurogenesis in sensory structures is associated with the type of stem cell present in the neurogenic niche of the zebrafish brain. *Eur. J. Neurosci.* 40 3591–3607. 10.1111/ejn.12729 25231569

[B103] Lindsey B. W. Hall Z. J. Heuzé A. Joly J. S. Tropepe V. Kaslin J. (2018). The role of neuro-epithelial-like and radial-glial stem and progenitor cells in development, plasticity, and repair. *Prog. Neurobiol.* 170 99–114. 10.1016/j.pneurobio.2018.06.004 29902500

[B104] Liu X. Wang Q. Haydar T. F. Bordey A. (2005). Nonsynaptic GABA signaling in postnatal subventricular zone controls proliferation of GFAP-expressing progenitors. *Nat. Neurosci.* 8 1179–1187. 10.1038/nn1522 16116450 PMC1380263

[B105] Llorens-Bobadilla E. Zhao S. Baser A. Saiz-Castro G. Zwadlo K. Martin-Villalba A. (2015). Single-cell transcriptomics reveals a population of dormant neural stem cells that become activated upon brain injury. *Cell Stem Cell* 17 329–340. 10.1016/j.stem.2015.07.002 26235341

[B106] Lugert S. Basak O. Knuckles P. Haussler U. Fabel K. Götz M. (2010). Quiescent and active hippocampal neural stem cells with distinct morphologies respond selectively to physiological and pathological stimuli and aging. *Cell Stem Cell* 6 445–456. 10.1016/j.stem.2010.03.017 20452319

[B107] Lugert S. Vogt M. Tchorz J. S. Müller M. Giachino C. Taylor V. (2012). Homeostatic neurogenesis in the adult hippocampus does not involve amplification of Ascl1 high intermediate progenitors. *Nat. Commun.* 3:670. 10.1038/ncomms1670 22334073

[B108] Lukaszewicz A. I. Nguyen C. Melendez E. Lin D. P. Teo J. L. Lai K. K. Y. (2019). The mode of stem cell division is dependent on the differential interaction of β-catenin with the kat3 coactivators CBP or p300. *Cancers* 11:962. 10.3390/cancers11070962 31324005 PMC6678591

[B109] Magnusson J. P. Göritz C. Tatarishvili J. Dias D. O. Smith E. M. K. Lindvall O. (2014). A latent neurogenic program in astrocytes regulated by Notch signaling in the mouse. *Science* 346 237–241. 10.1126/science.346.6206.237 25301628

[B110] Makantasi P. Dermon C. R. (2014). Estradiol treatment decreases cell proliferation in the neurogenic zones of adult female zebrafish (Danio Rerio) brain. *Neuroscience* 277 306–320. 10.1016/j.neuroscience.2014.06.071 25034512

[B111] Martynoga B. Mateo J. L. Zhou B. Andersen J. Achimastou A. Urbán N. (2013). Epigenomic enhancer annotation reveals a key role for NFIX in neural stem cell quiescence. *Genes Dev.* 27 1769–1786. 10.1101/gad.216804.113 23964093 PMC3759694

[B112] März M. Chapouton P. Diotel N. Vaillant C. Hesl B. Takamiya M. (2010a). Heterogeneity in progenitor cell subtypes in the ventricular zone of the zebrafish adult telencephalon. *Glia* 58 870–888. 20155821 10.1002/glia.20971

[B113] März M. Schmidt R. Rastegar S. Strähle U. (2010b). Expression of the transcription factor Olig2 in proliferating cells in the adult zebrafish telencephalon. *Dev. Dyn.* 239 3336–3349. 10.1002/dvdy.22455 20981834

[B114] März M. Schmidt R. Rastegar S. Strahle U. (2011). Regenerative response following stab injury in the adult zebrafish telencephalon. *Dev. Dyn.* 240 2221–2231. 10.1002/dvdy.22710 22016188

[B115] Matarredona E. R. Pastor A. M. (2019). Neural stem cells of the subventricular zone as the origin of human glioblastoma stem cells. Therapeutic implications. *Front. Oncol.* 9:779. 10.3389/fonc.2019.00779 31482066 PMC6710355

[B116] Mattar P. Ericson J. Blackshaw S. Cayouette M. (2015). A conserved regulatory logic controls temporal identity in mouse neural progenitors. *Neuron* 85 497–504. 10.1016/j.neuron.2014.12.052 25654255 PMC5912935

[B117] Menn B. Garcia-Verdugo J. M. Yaschine C. Gonzalez-Perez O. Rowitch D. Alvarez-Buylla A. (2006). Origin of oligodendrocytes in the subventricular zone of the adult brain. *J. Neurosci.* 26 7907–7918. 10.1523/jneurosci.1299-06.2006 16870736 PMC6674207

[B118] Merkle F. T. Fuentealba L. C. Sanders T. A. Magno L. Kessaris N. Alvarez-Buylla A. (2014). Adult neural stem cells in distinct microdomains generate previously unknown interneuron types. *Nat. Neurosci.* 17 207–214. 10.1038/nn.3610 24362763 PMC4100623

[B119] Merkle F. T. Mirzadeh Z. Alvarez-Buylla A. (2007). Mosaic organization of neural stem cells in the adult brain. *Science* 317 381–384. 10.1126/science.1144914 17615304

[B120] Mira H. Andreu Z. Suh H. Chichung Lie D. Jessberger S. Consiglio A. (2010). Signaling through BMPR-IA regulates quiescence and long-term activity of neural stem cells in the adult hippocampus. *Cell Stem Cell* 7 78–89. 10.1016/j.stem.2010.04.016 20621052

[B121] Mizrak D. Levitin H. M. Delgado A. C. Crotet V. Yuan J. Chaker Z. (2019). Single-cell analysis of regional differences in adult V-SVZ neural stem cell lineages. *Cell Rep.* 26 394–406. 30625322 10.1016/j.celrep.2018.12.044 PMC6368857

[B122] Moreno-Jiménez E. P. Flor-García M. Terreros-Roncal J. Rábano A. Cafini F. Pallas-Bazarra N. (2019). Adult hippocampal neurogenesis is abundant in neurologically healthy subjects and drops sharply in patients with Alzheimer’s disease. *Nat. Med.* 25 554–560. 10.1038/s41591-019-0375-9 30911133

[B123] Nait-Oumesmar B. Decker L. Lachapelle F. Avellana-Adalid V. Bachelin C. Baron-Van Evercooren A. (1999). Progenitor cells of the adult mouse subventricular zone proliferate, migrate and differentiate into oligodendrocytes after demyelination. *Eur. J. Neurosci.* 11 4357–4366. 10.1046/j.1460-9568.1999.00873.x 10594662

[B124] Nakatomi H. Kuriu T. Okabe S. Yamamoto S. Hatano O. Kawahara N. (2002). Regeneration of hippocampal pyramidal neurons after ischemic brain injury by recruitment of endogenous neural progenitors. *Cell* 110 429–441. 10.1016/s0092-8674(02)00862-0 12202033

[B125] Nelson B. Hodge R. Am Daza R. Tripathi P. Arnold S. Millen K. (2020). Intermediate progenitors support migration of neural stem cells into Dentate Gyrus outer neurogenic niches. *eLife* 9:e53777. 10.7554/elife.53777 32238264 PMC7159924

[B126] Obernier K. Cebrian-Silla A. Thomson M. Parraguez J. I. Anderson R. Guinto C. (2018). Adult neurogenesis is sustained by symmetric self-renewal and differentiation. *Cell Stem Cell* 22 221–234. 29395056 10.1016/j.stem.2018.01.003 PMC5802882

[B127] Ogino T. Sawada M. Takase H. Nakai C. Herranz-Pérez V. Cebrián-Silla A. (2016). Characterization of multiciliated ependymal cells that emerge in the neurogenic niche of the aged zebrafish brain. *J. Comp. Neurol.* 524 2982–2992. 10.1002/cne.24001 26991819

[B128] Ormerod B. K. Galea L. A. M. (2001). Reproductive status influences cell proliferation and cell survival in the dentate gyrus of adult female meadow voles: a possible regulatory role for estradiol. *Neuroscience* 102 369–379. 10.1016/s0306-4522(00)00474-7 11166123

[B129] Ormerod B. K. Lee T. T. Y. Galea L. A. M. (2003). Estradiol initially enhances but subsequently suppresses (via adrenal steroids) granule cell proliferation in the dentate gyrus of adult female rats. *J. Neurobiol.* 55 247–260. 10.1002/neu.10181 12672021

[B130] Ortega F. Gascón S. Masserdotti G. Deshpande A. Simon C. Fischer J. (2013). Oligodendrogliogenic and neurogenic adult subependymal zone neural stem cells constitute distinct lineages and exhibit differential responsiveness to Wnt signalling. *Nat. Cell Biol.* 15 602–613. 10.1038/ncb2736 23644466

[B131] Otsuki L. Brand A. H. (2018). Cell cycle heterogeneity directs the timing of neural stem cell activation from quiescence. *Science* 360 99–102. 10.1126/science.aan8795 29622651 PMC6538531

[B132] Paik J. H. Ding Z. Narurkar R. Ramkissoon S. Muller F. Kamoun W. S. (2009). FoxOs cooperatively regulate diverse pathways governing neural stem cell homeostasis. *Cell Stem Cell* 5 540–553. 10.1016/j.stem.2009.09.013 19896444 PMC3285492

[B133] Pardee A. B. (1974). A restriction point for control of normal animal cell proliferation. *Proc. Natl. Acad. Sci. U.S.A.* 71 1286–1290. 10.1073/pnas.71.4.1286 4524638 PMC388211

[B134] Pellegrini E. Mouriec K. Anglade I. Menuet A. Le Page Y. Gueguen M. M. (2007). Identification of aromatase-positive radial glial cells as progenitor cells in the ventricular layer of the forebrain in zebrafish. *J. Comp. Neurol.* 501 150–167. 10.1002/cne.21222 17206614

[B135] Pilz G. A. Bottes S. Betizeau M. Jörg D. J. Carta S. Simons B. D. (2018). Live imaging of neurogenesis in the adult mouse hippocampus. *Science* 359 658–662. 10.1126/science.aao5056 29439238 PMC6986926

[B136] Platel J. C. Angelova A. Bugeon S. Wallace J. Ganay T. Chudotvorova I. (2019). Neuronal integration in the adult mouse olfactory bulb is a non-selective addition process. *eLife* 8:e44830. 10.7554/eLife.44830 PMC6634973 31294694

[B137] Ponti G. Obernier K. Guinto C. Jose L. Bonfanti L. Alvarez-Buylla A. (2013). Cell cycle and lineage progression of neural progenitors in the ventricular-subventricular zones of adult mice. *Proc. Natl. Acad. Sci. U.S.A.* 110 E1045–E1054. 23431204 10.1073/pnas.1219563110 PMC3600494

[B138] Rai K. S. Hattiangady B. Shetty A. K. (2007). Enhanced production and dendritic growth of new dentate granule cells in the middle-aged hippocampus following intracerebroventricular FGF-2 infusions. *Eur. J. Neurosci.* 26 1765–1779. 10.1111/j.1460-9568.2007.05820.x 17883411

[B139] Raj B. Wagner D. E. McKenna A. Pandey S. Klein A. M. Shendure J. (2018). Simultaneous single-cell profiling of lineages and cell types in the vertebrate brain. *Nat. Biotechnol.* 36 442–450. 10.1038/nbt.4103 29608178 PMC5938111

[B140] Ramírez-Castillejo C. Sánchez-Sánchez F. Andreu-Agulló C. Ferrón S. R. Aroca-Aguilar J. D. Sánchez P. (2006). Pigment epithelium-derived factor is a niche signal for neural stem cell renewal. *Nat. Neurosci.* 9 331–339. 10.1038/nn1657 16491078

[B141] Rastegar S. Parimisetty A. Cassam Sulliman N. Narra S. S. Weber S. Rastegar M. (2019). Expression of adiponectin receptors in the brain of adult zebrafish and mouse: links with neurogenic niches and brain repair. *J. Comp. Neurol.* 527 2317–2333. 30843204 10.1002/cne.24669

[B142] Reimer M. M. Kuscha V. Wyatt C. Sorensen I. Frank R. E. Knuwer M. (2009). Sonic hedgehog is a polarized signal for motor neuron regeneration in adult Zebrafish. *J. Neurosci.* 29 15073–15082. 10.1523/jneurosci.4748-09.2009 19955358 PMC2841428

[B143] Renault V. M. Rafalski V. A. Morgan A. A. Salih D. A. M. Brett J. O. Webb A. E. (2009). FoxO3 regulates neural stem cell homeostasis. *Cell Stem Cell* 5 527–539. 10.1016/j.stem.2009.09.014 19896443 PMC2775802

[B144] Roccio M. Schmitter D. Knobloch M. Okawa Y. Sage D. Lutolf M. P. (2013). Predicting stem cell fate changes by differential cell cycle progression patterns. *Development* 140 459–470. 10.1242/dev.086215 23193167

[B145] Rochefort C. Gheusi G. Vincent J. D. Lledo P. M. (2002). Enriched odor exposure increases the number of newborn neurons in the adult olfactory bulb and improves odor memory. *J. Neurosci.* 22 2679–2689. 10.1523/jneurosci.22-07-02679.2002 11923433 PMC6758329

[B146] Rodríguez F. López J. C. Vargas J. P. Gómez Y. Broglio C. Salas C. (2002). Conservation of spatial memory function in the pallial forebrain of reptiles and ray-finned fishes. *J. Neurosci.* 22 2894–2903. 10.1523/jneurosci.22-07-02894.2002 11923454 PMC6758289

[B147] Rodriguez-Viales R. Diotel N. Ferg M. Armant O. Eich J. Alunni A. (2015). The helix-loop-helix protein Id1 controls stem cell proliferation during regenerative neurogenesis in the adult zebrafish telencephalon. *Stem Cells* 33 892–903. 10.1002/stem.1883 25376791

[B148] Rothenaigner I. Krecsmarik M. Hayes J. A. Bahn B. Lepier A. Fortin G. (2011). Clonal analysis by distinct viral vectors identifies bona fide neural stem cells in the adult zebrafish telencephalon and characterizes their division properties and fate. *Development* 138 1459–1469. 10.1242/dev.058156 21367818

[B149] Sato Y. Yano H. Shimizu Y. Tanaka H. Ohshima T. (2017). Optic nerve input-dependent regulation of neural stem cell proliferation in the optic tectum of adult zebrafish. *Dev. Neurobiol.* 77 474–482. 10.1002/dneu.22423 27480480

[B150] Schäffner I. Minakaki G. Khan M. A. Balta E. A. Schlötzer-Schrehardt U. Schwarz T. J. (2018). FoxO function is essential for maintenance of autophagic flux and neuronal morphogenesis in adult neurogenesis. *Neuron* 99 1188–1203. 30197237 10.1016/j.neuron.2018.08.017 PMC6186958

[B151] Seri B. García-Verdugo J. M. Collado-Morente L. McEwen B. S. Alvarez-Buylla A. (2004). Cell types, lineage, and architecture of the germinal zone in the adult dentate gyrus. *J. Comp. Neurol.* 478 359–378. 10.1002/cne.20288 15384070

[B152] Shah P. T. Stratton J. A. Stykel M. G. Abbasi S. Sharma S. Mayr K. A. (2018). Single-cell transcriptomics and fate mapping of ependymal cells reveals an absence of neural stem cell function. *Cell* 173 1045–1057. 29727663 10.1016/j.cell.2018.03.063

[B153] Shin Y. J. Park S. K. Jung Y. J. Kim Y. N. Kim K. S. Park O. K. (2015). Nanobody-targeted E3-ubiquitin ligase complex degrades nuclear proteins. *Sci. Rep.* 5:14269. 10.1038/srep14269 PMC4571616 26373678

[B154] Simons B. D. Clevers H. (2011). Strategies for homeostatic stem cell self-renewal in adult tissues. *Cell* 145 851–862. 10.1016/j.cell.2011.05.033 21663791

[B155] Sirko S. Behrendt G. Johansson P. A. Tripathi P. Costa M. Bek S. (2013). Reactive glia in the injured brain acquire stem cell properties in response to sonic hedgehog glia. *Cell Stem Cell* 12 426–439. 10.1016/j.stem.2013.01.019 23561443

[B156] Skaggs K. Goldman D. Parent J. M. (2014). Excitotoxic brain injury in adult zebrafish stimulates neurogenesis and long-distance neuronal integration. *Glia* 62 2061–2079. 10.1002/glia.22726 25043622 PMC4205181

[B157] Sohn J. Orosco L. Guo F. Chung S. H. Bannerman P. Ko E. M. (2015). The subventricular zone continues to generate corpus callosum and rostral migratory stream astroglia in normal adult mice. *J. Neurosci.* 35 3756–3763. 10.1523/jneurosci.3454-14.2015 25740506 PMC6605576

[B158] Song H. Berg D. A. Bond A. M. Ming G. (2018). Radial glial cells in the adult dentate gyrus: What are they and where do they come from? *F1000Res.* 7:277. 10.12688/f1000research.12684.1 29568500 PMC5840617

[B159] Song J. Zhong C. Bonaguidi M. A. Sun G. J. Hsu D. Gu Y. (2012). Neuronal circuitry mechanism regulating adult quiescent neural stem-cell fate decision. *Nature* 489 150–154. 10.1038/nature11306 22842902 PMC3438284

[B160] Spassky N. Merkle F. T. Flames N. Tramontin A. D. Garcia-Verdugo J. M. Alvarez-Buylla A. (2005). Adult ependymal cells are postmitotic and are derived from radial glial cells during embryogenesis. *J. Neurosci.* 25 10–18. 10.1523/jneurosci.1108-04.2005 15634762 PMC6725217

[B161] Spencer S. L. Cappell S. D. Tsai F. C. Overton K. W. Wang C. L. Meyer T. (2013). The proliferation-quiescence decision is controlled by a bifurcation in CDK2 activity at mitotic exit. *Cell* 155 369–383. 10.1016/j.cell.2013.08.062 24075009 PMC4001917

[B162] Sueda R. Imayoshi I. Harima Y. Kageyama R. (2019). High Hes1 expression and resultant Ascl1 suppression regulate quiescent vs. active neural stem cells in the adult mouse brain. *Genes Dev.* 33 511–523. 10.1101/gad.323196.118 30862661 PMC6499325

[B163] Suh H. Consiglio A. Ray J. Sawai T. D’Amour K. A. Gage F. H. (2007). *In vivo* fate analysis reveals the multipotent and self-renewal capacities of Sox2+ neural stem cells in the adult hippocampus. *Cell Stem Cell* 1 515–528. 10.1016/j.stem.2007.09.002 18371391 PMC2185820

[B164] Tanapat P. Hastings N. B. Gould E. (2005). Ovarian steroids influence cell proliferation in the dentate gyrus of the adult female rat in a dose- and time-dependent manner. *J. Comp. Neurol.* 481 252–265. 10.1002/cne.20385 15593136

[B165] Tanapat P. Hastings N. B. Reeves A. J. Gould E. (1999). Estrogen stimulates a transient increase in the number of new neurons in the dentate gyrus of the adult female rat. *J. Neurosci.* 19 5792–5801. 10.1523/jneurosci.19-14-05792.1999 10407020 PMC6783062

[B166] Taverna E. Huttner W. B. (2019). “The Golgi apparatus in polarized neuroepithelial stem cells and their progeny: canonical and noncanonical features,” in *Results and Problems in Cell Differentiation*, ed. Kloc M. (Berlin: Springer Verlag), 359–375. 10.1007/978-3-030-23173-6_15 31435803

[B167] Tea J. Alderman S. L. Gilmour K. M. (2019). Social stress increases plasma cortisol and reduces forebrain cell proliferation in subordinate male zebrafish (Danio rerio). *J. Exp. Biol.* 222:jeb194894. 10.1242/jeb.194894 30530837

[B168] Than-Trong E. Bally-Cuif L. (2015). Radial glia and neural progenitors in the adult zebrafish central nervous system. *Glia* 63 1406–1428. 10.1002/glia.22856 25976648

[B169] Than-Trong E. Kiani B. Dray N. Ortica S. Simons B. Rulands S. (2020). Lineage hierarchies and stochasticity ensure the long-term maintenance of adult neural stem cells. *Sci. Adv.* 6:eaaz5424. 10.1126/sciadv.aaz5424 32426477 PMC7190328

[B170] Than-Trong E. Ortica-Gatti S. Mella S. Nepal C. Alunni A. Bally-Cuif L. (2018). Neural stem cell quiescence and stemness are molecularly distinct outputs of the Notch3 signalling cascade in the vertebrate adult brain. *Development* 145:dev161034. 10.1242/dev.161034 29695612 PMC6001379

[B171] Toda T. Parylak S. L. Linker S. B. Gage F. H. (2019). The role of adult hippocampal neurogenesis in brain health and disease. *Mol. Psychiatry* 24 67–87. 10.1038/s41380-018-0036-2 29679070 PMC6195869

[B172] Topp S. Stigloher C. Komisarczuk A. Z. Adolf B. Becker T. S. Bally-Cuif L. (2008). Fgf signaling in the zebrafish adult brain: association of Fgf activity with ventricular zones but not cell proliferation. *J. Comp. Neurol.* 510 422–439. 10.1002/cne.21802 18666124

[B173] Tozer S. Baek C. Fischer E. Goiame R. Morin X. (2017). Differential routing of mindbomb1 via centriolar satellites regulates asymmetric divisions of neural progenitors. *Neuron* 93 542–551.e4. 10.1016/j.neuron.2016.12.042 28132826

[B174] Turrero García M. Chang Y. Arai Y. Huttner W. B. (2016). S-phase duration is the main target of cell cycle regulation in neural progenitors of developing ferret neocortex. *J. Comp. Neurol.* 524 456–470. 10.1002/cne.23801 25963823 PMC5008145

[B175] Unger M. S. Marschallinger J. Kaindl J. Höfling C. Rossner S. Heneka M. T. (2016). Early changes in hippocampal neurogenesis in transgenic mouse models for Alzheimer’s Disease. *Mol. Neurobiol.* 53 5796–5806. 10.1007/s12035-016-0018-9 27544234 PMC5012146

[B176] Urbán N. van den Berg D. L. C. Forget A. Andersen J. Demmers J. A. A. Hunt C. (2016). Return to quiescence of mouse neural stem cells by degradation of a proactivation protein. *Science* 353 329–340. 10.1126/science.aaf4802 PMC5321528 27418510

[B177] Van Praag H. Christie B. R. Sejnowski T. J. Gage F. H. (1999). Running enhances neurogenesis, learning, and long-term potentiation in mice. *Proc. Natl. Acad. Sci. U.S.A.* 96 13427–13431. 10.1073/pnas.96.23.13427 10557337 PMC23964

[B178] von Trotha J. W. Vernier P. Bally-Cuif L. (2014). Emotions and motivated behavior converge on an amygdala-like structure in the zebrafish. *Eur. J. Neurosci.* 40 3302–3315. 10.1111/ejn.12692 25145867 PMC4278443

[B179] Webb A. Pollina E. Vierbuchen T. Urbán N. Ucar D. Leeman D. (2013). FOXO3 shares common targets with ASCL1 genome-wide and inhibits ASCL1-dependent neurogenesis. *Cell Rep.* 4 477–491. 10.1016/j.celrep.2013.06.035 23891001 PMC3838667

[B180] Wu C. W. Chang Y. T. Yu L. Chen H. I. Jen C. J. Wu S. Y. (2008). Exercise enhances the proliferation of neural stem cells and neurite growth and survival of neuronal progenitor cells in dentate gyrus of middle-aged mice. *J. Appl. Physiol.* 105 1585–1594. 10.1152/japplphysiol.90775.2008 18801961

[B181] Xing Y. L. Röth P. T. Stratton J. A. S. Chuang B. H. A. Danne J. Ellis S. L. (2014). Adult neural precursor cells from the subventricular zone contribute significantly to oligodendrocyte regeneration and remyelination. *J. Neurosci.* 34 14128–14146. 10.1523/jneurosci.3491-13.2014 25319708 PMC6705285

[B182] Zambusi A. Ninkovic J. (2020). Regeneration of the central nervous system-principles from brain regeneration in adult zebrafish. *World J. Stem Cells* 12 8–24. 10.4252/wjsc.v12.i1.8 32110272 PMC7031763

[B183] Zhang G. Ferg M. Lübke L. Takamiya M. Beil T. Gourain V. (2020). Bone morphogenetic protein signaling regulates Id1-mediated neural stem cell quiescence in the adult zebrafish brain via a phylogenetically conserved enhancer module. *Stem Cells.* 10.1002/stem.3182 [Epub ahead of print]. 32246536

[B184] Zhang R. Boareto M. Engler A. Louvi A. Giachino C. Iber D. (2019). Id4 downstream of Notch2 maintains neural stem cell quiescence in the adult hippocampus. *Cell Rep.* 28 1485–1498. 31390563 10.1016/j.celrep.2019.07.014

[B185] Zhao M. Li D. Shimazu K. Zhou Y. X. Lu B. Deng C. X. (2007). Fibroblast growth factor receptor-1 is required for long-term potentiation, memory consolidation, and neurogenesis. *Biol. Psychiatry* 62 381–390. 10.1016/j.biopsych.2006.10.019 17239352

